# Bacterial biofilm colonization and succession in tropical marine waters are similar across different types of stone materials used in seawall construction

**DOI:** 10.3389/fmicb.2022.928877

**Published:** 2022-07-25

**Authors:** Stephen Summers, Y. Shona Pek, Deepthi P. Vinod, Diane McDougald, Peter A. Todd, William R. Birch, Scott A. Rice

**Affiliations:** ^1^Singapore Centre for Environmental Life Sciences Engineering, Nanyang Technological University, Singapore, Singapore; ^2^Institute of Materials Research and Engineering, Agency for Science, Technology and Research (A*STAR), Singapore, Singapore; ^3^School of Bioscience and Technology, Vellore Institute of Technology, Vellore, India; ^4^Australian Institute for Microbiology and Infection, The University of Technology Sydney, Sydney, NSW, Australia; ^5^Department of Biological Sciences, National University of Singapore, Singapore, Singapore; ^6^Agriculture and Food, Microbiomes for One Systems Health, Commonwealth Scientific and Industrial Research Organisation (CSIRO), Canberra, ACT, Australia

**Keywords:** seawall, biofilm, microbial diversity, marine microbes, coastal protection

## Abstract

Seawalls are important in protecting coastlines from currents, erosion, sea-level rise, and flooding. They are, however, associated with reduced biodiversity, due to their steep orientation, lack of microhabitats, and the materials used in their construction. Hence, there is considerable interest in modifying seawalls to enhance the settlement and diversity of marine organisms, as microbial biofilms play a critical role facilitating algal and invertebrate colonization. We assessed how different stone materials, ranging from aluminosilicates to limestone and concrete, affect biofilm formation. Metagenomic assessment of marine microbial communities indicated no significant impact of material on microbial diversity, irrespective of the diverse surface chemistry and topography. Based on KEGG pathway analysis, surface properties appeared to influence the community composition and function during the initial stages of biofilm development, but this effect disappeared by Day 31. We conclude that marine biofilms converged over time to a generic marine biofilm, rather than the underlying stone substrata type playing a significant role in driving community composition.

## Introduction

A warming planet has led to thermal expansion of the oceans and melting of ice sheets, thus raising sea levels around the world (Warrick and Oerlemans, 1990; Douglas, 1991; Golledge et al., 2019) and accelerating coastal erosion and flooding. Low lying islands and coastal cities are particularly vulnerable (Bruun, 1962; Nicholls and Cazenave, 2010). As human populations continue to migrate toward the coast, the risk to property and life will increase (Temmerman et al., 2013). The installation of artificial defense structures such as seawalls has become the primary mitigation strategy for preventing some of the more extreme effects of sea level rise (Firth et al., 2014; Todd et al., 2019). These can be constructed from any material that can withstand the constant action of waves and currents, but concrete and natural stone materials are commonly used (Dugan et al., 2011). For many countries, large scale installations represent a significant amount of the coastline. For example, Korea’s artificial Saemangeum dyke is > 33 km in length (Lam et al., 2009), Japan is planning over 400 km of seawalls to protect from future tsunami disasters (Yamashita, 2020) and in Singapore, > 63% of the coast is some form of seawall, either vertical concrete or granite rip-rap (Lai et al., 2015). However, seawall proliferation comes at a considerable cost to coastal ecosystems. Heatherington and Bishop (2012) reported that seawalls affect the physical and hydrodynamic processes of a natural coastal region, such as sediment and larval transportation. Others have shown that they support lower species diversity and different communities (Ming et al., 2010; Lai et al., 2015) and can encourage invasive species (Simkanin et al., 2012). Critically, the pre-existing habitat is often obliterated by the installation of such large and intrusive infrastructure (Firth et al., 2014).

While there exists a strong ecological case for moving away from seawalls to ecosystem-based coastal defenses such as mangroves and saltmarshes, these require substantial shoreline space that is generally not available near cities (Temmerman et al., 2013). It is inevitable that seawalls will continue to be constructed. However, there remains the potential to improve their design to make them more viable as a habitat for marine life (Loke et al., 2015; Morris et al., 2019). One solution that is being extensively researched is to increase the topographical complexity of the seawall to provide niche availability for a wider range of species (Loke et al., 2019). It is thought that anthropogenic seawall installations are lacking in complexity and thus have a reduce niche environment (Lawrence et al., 2021). However, more recent investigations have highlighted that complexity alone is not universally positive with respect to increasing biodiversity, as this can be affected by local stressors, such as predation and should be seen only as an element in improving seawalls (Chee et al., 2021; Strain et al., 2021). Another, much less studied approach, is to identify construction materials that may encourage colonization by marine biota (Dodds et al., 2022; Hartanto et al., 2022). Before any invertebrates or other eukaryotic organisms settle on the seawall surface, it is known that microbial colonizers begin to form “conditioning” biofilms. These biofilms are a vital first step in the recruitment of other organisms (Freckelton et al., 2017), as the biofilms and species therein have been shown to have a role in regulating the settlement of sessile organisms onto the substratum (Hadfield, 2011; Freckelton et al., 2022). However, it is unclear whether manipulating the seawall construction material has any substantial impact on the biofilms that initially form on the seawall surface. Studies comparing seawall microbial biofilms to those found on natural rocky shorelines have shown differences in the relative abundance of biofilm inhabitants between the two environments (Tan et al., 2015). However, settlement control plates present at both sites (seawall and rocky shore) did not differ in biofilm settlement. This suggests that the locations are not driving any observed differences, but instead it is the chemical and/or physical properties of those sites that drive differences in the settled and formed microbial communities.

The current study selected natural stones comprising aluminosilicates and calcium carbonate, which present different surface chemistry. As substrates, they also differ in their resistance to erosion. Granite is an aluminosilicate that resists chemical erosion, which has led to its widespread implementation as a durable mechanical barrier on seawalls. In contrast, limestone is composed from calcium carbonate, which is more readily eroded in a mildly acidic environment (Revelle and Emery, 1957), thus giving rise to natural macrostructures with higher complexity (Higgins, 1980). Sandstone differs by offering a high degree of porosity and it has been reported to promote the growth of foliose algae (Green et al., 2012). Settlement on natural stones and the ensuing erosion generated by microbial species have been studied and shown to influence the microbial biofilms associated with the stones (Cockell et al., 2011; Summers et al., 2016). Differences have been observed between microorganisms weathering limestone, granite, and concrete (Coombes et al., 2011), with boring also being influenced by environmental factors (Cockell and Herrera, 2008). The surface chemistry of aluminosilicates presents silanol groups that have high affinity to water (Birch et al., 2008), while calcium carbonate substrates present moieties that are reported to bind to organic molecules (Suess, 1970; Carter, 1978; Zeller et al., 2020).

Based on these observations, we hypothesized that differing construction materials would recruit different microbial biofilm communities. If this hypothesis is correct, then construction material could be a key component not only for seawall strength and durability, but also for any biodiversity enhancement strategy, such as the addition of tiles or cladding. Therefore, to address the role of substrata in marine biofilm community composition, we examined several construction materials for the formation of natural tropical marine biofilms, to elucidate what impacts these varied materials may have on the initial colonization of an anthropogenic sea defense structures.

## Materials and methods

### Substrata used as proxies for potential seawall materials

To determine the effect that stone type has on marine biofilm formation on a tropical seawall, eight different substrata to monitor biofilm community formation were selected to represent salient potential construction materials, encompassing aluminosilicates and calcium carbonates, respectively. These were: granite (GRANITE), limestone (LIMESTN), marble (MARBLE), pink sandstone (SANDSP), violet sandstone (SANDSV), pH neutralized concrete (CONCTN), rough textured concrete (CONCRO), and smooth textured concrete (CONCTR). These were chosen to represent salient natural stone categories, with differing surface structure, mineral, and chemical compositions. They were selected with uniform surface appearance, on a cm-scale, purchased from a local stone mason (Hot Spring Stone Pte. Ltd., Singapore). The surfaces were saw-cut without polishing, thus mitigating surface roughness variations and avoiding alteration of their surface chemistry, respectively. Stone samples were used as 5 cm × 5 cm × 2.5 cm coupons and concrete coupons were prepared by pouring concrete premix into set molds of the same dimensions. Concrete grain composition was 80% sand and 20% cement, resulting in a mortar comparable to the standards employed in masonry. The intrinsic alkalinity of as-formed concrete, with pH ≈ 13, can be mitigated by carbonation. This was achieved by exposing the concrete coupons to a saturated CO_2_ environment, generated within a sealed vessel by sublimating dry ice, for 1 month (Hsiung et al., 2020). This process confers pH neutrality to the surface region, and progressively penetrates into the bulk volume of the concrete.

The stone surfaces were examined by scanning electron microscopy (SEM) using a JEOL JSM-7600F field emission scanning electron microscope operating at an accelerating voltage of 15 kV. Each was sputter coated with gold using a JEOL JFC-1200 fine coater and its surface morphology was imaged, together with an elemental cartography. Surface elemental and compositional analysis was performed with an Oxford Energy Dispersive X-Ray Analysis (EDX) system. Internal surface area, pore volume, and overall porosity measurements were obtained via Brunauer–Emmett–Teller (BET) nitrogen adsorption, using a Micromeritics ASAP 2020 system. Pore distribution was obtained from mercury intrusion, using a MicroActive Autopore V9600 mercury porosimeter. Average surface roughness (Ra) was measured over a 5 mm length using a TENCOR P-10 Surface Profiler at a scan speed of 50 μms^–1^, with a sampling rate of 100 Hz. Thermal conductivity was measured with a NETZSCH HFM 436 heat flow meter.

### Natural biofilm generation— Amplicon sequencing experiment

To develop natural marine biofilms on the different stone types, each coupon was sterilized in an autoclave (121°C for 15 min), submerged vertically in one of two outdoor flow-through aquaria to mitigate sedimentation and were supplied with a constantly renewed supply of sand filtered natural seawater for 1 month. Each aquarium held 300 L of seawater and was fed at a flow rate of 15 L min^–1^, resulting in three water exchanges per hour. This exchange rate was chosen to mitigate any effects from leachates from the stone coupons during the incubation. Temperature and LUX intensity logs of the water and air (<5 cm above water line) were recorded each hour for 31 days, using Hobo data loggers (Onset, 8k data logger pendant).

To monitor biofilm development over the 1-month period, sacrificial coupons of all eight substrata types were removed at six time points (after 3 h and 1, 3, 7, 14, and 31 days) for examination. The experiment was divided over two aquaria, fed from the same seawater supply, with two replicates of each treatment were present in each tank, for a total of four replicates overall for each stone type at each time point (192 coupons in total). The position of each coupon within each aquarium was randomized, using the “sample” function in R base, to mitigate any positional bias within the aquaria with respect to biofilm growth. To determine the variability of the microbial community in the sand filtered seawater, 50 mL of seawater was collected at each time point to act as a background control. These aliquots were filtered onto a 0.22 μm polycarbonate filter and subsequently stored at −20°C.

### Natural biofilm generation—Metagenome sequencing experiment

This experiment was repeated 12 months later using a similar experimental design to that employed for amplicon sequencing. The second run did not include concrete coupons and sacrificial sampling occurred weekly (weeks 1, 2, 3, and 4). One reason for omitting the concrete coupons was the emergence from our trials with chemically altered concretes that their differences appear to have little effect on biological diversity at the macro scale (Hsiung et al., 2020). Glass microscope slides were also included during this experiment to allow comparison with other studies which employ glass substrata as controls. All analyses involving the control glass slides were standardized to account for the difference in area between the glass and stone substrata. In addition, total nitrogen (TN), total organic carbon (TOC), and total carbon (TC) from the aquaria were measured each week for the duration of the experiment (TOC-L analyzer, Shimadzu, Singapore), from these data the total inorganic carbon (TIC) was calculated. The water chemistry was recorded each week using a YSI ProQuatro meter (YSI; Ohio, United States). The primary purpose of this second experiment was to enable metagenomic sequencing of the biofilms. All treatments were performed in triplicate for this experiment.

### Biofilm collection and nucleic acid extraction

To assess the biodiversity of the biofilms across time, the stone coupons were removed from the aquaria at the time points indicated above. The stones were removed from the aquaria and the biofilm sampled from a single 5 × 5 cm face. A sterile cotton swab was used to collect the biofilm material formed on the surface. These were immediately labeled and frozen at −20°C, before being transported to the laboratory in a liquid nitrogen cooled dry shipper and then stored at −20°C until nucleic acid extraction was performed.

To extract and isolate the nucleic acids, individual cotton swab heads were removed and placed into a Lysis Matrix E tube, before extraction with the FastDNA soil extract kit (MP Biomedical, Singapore), which was implemented according to the manufacturer’s instructions. The resulting total nucleic acid extractions were quantified using a NanoDrop spectrophotometer, before being stored at −20°C for downstream analysis. These concentration data were employed as proxies for the general biomass of the biofilms and cannot distinguish between the main taxonomic groups (Bohórquez et al., 2017). For the background controls, the seawater was filtered and each filter was cut into half and placed in a sterile microcentrifuge tube, before being frozen in liquid N_2_ and then milled down to a powder, which was then subjected to the same process of extraction as the cotton swabs. All extractions were stored at −20°C, for downstream analysis.

### Nucleic acid amplification and MiSeq amplicon library preparation

Two sets of primers were used to target the 16S rRNA (515f-806r; V4 region bacteria and archaea) (Caporaso et al., 2011) and the 18S rRNA (Euk 1392f—Euk Br; V9 region) (Medlin et al., 1988; Lane, 1991) to generate libraries for amplicon sequencing. For each reaction for the 16S rRNA gene, 0.5 μL of each primer (10 μM) was added to 10 μL of Jumpstart Taq Ready Mix (#P2893-100RXN, Merck, Singapore). Additionally, 1 μL of gDNA template was added to each reaction and then made up to 20 μL using sdH_2_O. For each reaction for the 18S rRNA gene, 1.0 μL of each primer (10 μM) was added to 12 μL of Jumpstart Taq Ready Mix. Additionally, 1 μL of gDNA template was added to each reaction and made up to 25 μL using sdH_2_O. The amplification condition for the 16S rRNA libraries were 94°C for 3 min initial denaturation, followed by 35 cycles of 94°C (45 s), 53°C (60 s), and 72°C (90 s); concluding with 72°C for 10 min. Amplification of the 18S rRNA libraries was achieved by 94°C for 3 min initial denaturation, followed by 35 cycles of 94°C (45 s), 57°C (60 s), and 72°C (90 s), concluding with 72°C for 10 min. Upon amplification, sequencing libraries were generated using the TruSeq kit v2.0 and paired-end MiSeq sequencing was conducted.

### Amplicon sequence quality, assembly and identification

Quality filtration of the raw sequences was performed using the DaDa2 (Callahan et al., 2016) protocol, within an R environment (R Core Team, 2014). Briefly, all sequences were observed for QC Phred scores above 25 and bases trimmed from the start (10 bp) and end (50 bp) of the fragments. Subsequently, these sequences were assembled and processed using DaDa2 to obtain chimeric free amplicon single nucleotide variants (SNVs). Prokaryotic and eukaryotic sequences were identified through comparison to the Silva v.132 Ribosomal database. Each identified SNV required a minimum similarity of 80% in order to be classified as a particular taxon, otherwise it was labeled as unclassified. Rarefaction plots indicating sampling effort are available (Supplementary Figure 1).

### Metagenome sequencing library

Total nucleic acid extractions from the second experiment were quantified using a Qubit HS (high sensitivity) dsDNA kit. Sequencing library preparation was achieved using the Swift Biosciences Accel-NGS 2S Plus DNA Kit, as per the manufacturer’s instructions. Using a Covaris S220 ultrasonicator, fragments were sheared to ∼450 bp, prior to adding barcodes using the Swift Biosciences 2S dual indexing kit. The subsequent libraries were checked for fragment length using a Bioanalyser DNA 7500 chip and quantification was performed using a picrogreen fluorescence assay. Libraries were then standardized to 4 nM as verified by qPCR (Kapa Biosystems Library Quantification kit, Applied Biosciences). An equimolar pool of all samples was generated and sequenced on Illumina HiSeq 2500 rapid runs (10–11 pM: V2 rapid sequencing chemistry), yielding reads of 251 bp paired-end sequences.

### Metagenomic sequence analyses

Shotgun sequencing reads were assessed for quality and adapters were removed using the Trim Galore package (v0.6.4_dev). Low quality ends were removed from sequences with a phred score lower than 20. Paired reads were merged using Pear (v0.9.6), using default settings. The resulting sequences were trimmed to between 200 and 300 bases before being randomly sub-sampled, which resulted in 750,000 reads per sample. All sub-sampled reads were subjected to taxonomic identification by comparison to the Maxikraken2 database (Loman, 2020), using the Kraken2 package (v2.0.8_beta) and visualized as Sankey plots using R (Pavian Package). Independent of the taxonomic identification, the raw reads were *de novo* assembled using spades (v3.13.0), thus generating contigs. Gene annotation of the contigs was conducted using Prokka (1.13) and these were examined for functional identification using Microbeannotator (v2.0.5) which annotated the proteins against the KOfam and RefSeq databases, before being searches and compared again the KEGG database (release 551) using the KOfamscan package (Kanehisa et al., 2016; Kanehisa and Sato, 2019). From these metagenomic sequencing reads, all rRNA reads were identified and classified using sortmerna package (v4.3.4) and Kraken2 (v2.0.8_beta) package, both equipped with the Silva v.132 database used for the amplicon experiments. Rarefaction plots indicating sampling effort of all rRNAs are available (Supplementary Figure 3).

### Statistical analysis of results

Data were processed and visualized in R Core Team (2014) using the vegan (Oksanen et al., 2011), complexheatmap (Gu et al., 2016), and ggplots (Wickham, 2016). Briefly, all absolute values of abundance were standardized to relative abundance for SNVs. Statistical analysis was performed using Permanova (Anderson, 2001) (adonis; vegan) to determine community differences and univariate differences between data were assessed using ANOVA or ANCOVA analyses (aov; vegan). Potential functional differences were assessed using ANOSIM to determine if identified complete KEGG pathways differed between treatment types. *Post hoc* analyses were conducted using the Tukey test (Tukey, 1949) of difference, to determine variables that differ significantly (*p* ≤ 0.05). Alpha-diversity was assessed using Shannon-Weiner indices (Shannon, 1948). All data was arcsine transformed prior to statistical assessment, to ensure the assumptions needed for parametric analyses were met where appropriate.

### Data availability

Data generated during this study is available within the Supplementary Information. All sequences are available from the NCBI, Sequence Read Archive (BioProject: PRJNA698754).

## Results

### Biofilm physico-chemical environment

Three of the five natural stone substrata examined were classed as aluminosilicates (Al_2_SiO_5_; SANDSP, SANDSV, and GRANITE), while the remaining were calcium carbonates (LIMESTN and MARBLE). The elemental composition of LIMESTN was predominately Ca, C, and O, with trace amounts of Mg (0.02%) and Ni (0.1%) (Table 1). While broadly similar to LIMESTN, MARBLE contained a lower fraction of Ca, together with higher percentage (11.5%) of Mg and trace amounts of Co and Al. SANDSP, SANDSV, and GRANITE contained Si and Al, as anticipated for aluminosilicates, with higher fractions of Mg in SANDSV (3.64%) and GRANITE (1.72%). In GRANITE, Fe generally appeared to be co-localized with Mg. Al and Na were similarly co-located, but in complementary locations (Supplementary Figure 3). A similar trend was observed for Al and Na, in SANDSP. SANDSV presented comparatively small, localized regions of Ca, which are presumed to indicate the presence of incorporated calcium carbonate fragments. Si and O, associated with silica moieties, were exhibited uniformly over the natural stone aluminosilicates. In contrast, CONCRO samples showed Si in locations complementary to Ca.

**TABLE 1:** Surface elemental composition of the treatment stone types obtained from SEM/EDX.

Substratum	Ca (%)	C (%)	Al (%)	Si (%)	O (%)	Fe (%)	Mg (%)	Na (%)	K (%)	Ti (%)	Ni (%)	Co (%)	S (%)
LIMESTN	41.62 (1.27)	13.06 (0.68)			45.18 (0.57)		0.02 (0.05)				0.10 (0.14)		
MARBLE	21.52 (1.25)	17.18 (0.38)	0.04 (0.09)		49.56 (1.04)		11.5 (0.45)					0.12 (0.08)	
SANDSP	0.32 (0.05)	8.96 (0.37)	3.28 (0.26)	35.06 (0.65)	48.32 (0.65)	0.56 (0.06)	0.30 (0.10)	0.04 (0.06)	3.08 (0.34)	0.06 (0.13)			
SANDSV	1.38 (0.08)	8.28 (0.85)	6.62 (0.23)	24.00 (0.50)	45.02 (0.91)	5.66 (0.23)	3.64 (0.39)	3.48 (0.19)	1.58 (0.18)	0.28 (0.05)	0.04 (0.06)	0.02 (0.05)	
GRANITE	6.72 (1.08)	9.90 (1.59)	10.26 (1.35)	22.42 (2.31)	37.76 (4.13)	7.82 (5.43)	1.72 (1.42)	1.88 (0.37)	0.40 (0.24)	0.62 (1.39)	0.10 (0.14)	0.06 (0.06)	0.06 (0.06)
CONCRO	14.2 (5.44)	10.7 (3.18)	1.4 (0.07)	20.6 (8.49)	51.4 (5.73)	0.7 (0.21)	0.3 (0.11)		0.2 (0.07)				0.4 (0.28)
CONCTR	33.16 (1.11)	13.46 (1.63)	0.82 (0.18)	3.38 (1.94)	45.05 (3.62)	0.72 (0.04)	0.74 (0.42)	0.22 (0.20)	0.42 (0.33)			0.03 (0.06)	0.2 (0.28)
CONCTN	25.47 (0.80)	20.30 (2.31)	0.90 (0.26)	1.7 (0.3)	48.67 (2.84)	0.63 (0.25)	0.73 (0.38)	0.27 (0.12)	0.23 (0.15)			0.10 (0.01)	0.3 (0.1)

Each element has been listed as the w/w percentage fraction of the sum of the detected elements. Abbreviations are used in subsequent tables and figures to indicate each stone type. Values presented indicate the measured mean with standard deviations in parentheses.

While natural stone coupons were cut with identical tooling, their surfaces exhibited different roughness. This is attributed to differences in hardness and their exposed porosity. The structure of LIMESTN presents a fractional pore volume over 11% (Table 2), with a larger fraction in the sub-micron range ≈280–500 nm and a smaller proportion over ≈50–250 μm. This differs substantially from MARBLE, which presents a 10-fold lower overall porosity (≈ 1%) that occurs primarily around 250 nm and over a larger size range that is similar to LIMESTN ≈65–270 μm. These ranges were estimated from the width at half of the maximum of pore size (Supplementary Figure 2). SANDSP and SANDSV both present fractional pore volumes of the same order as limestone, with a significant proportion occurring in the range 150–500 nm. They contrast in their larger pore dimensions, with SANDSP exhibiting a narrow peak around 7.9 μm, while SANDSV offers a broad distribution, from ≈30 to 300 μm. GRANITE offers a comparatively closed structure, with a fractional pore volume that is similar to MARBLE. This appears as a broad distribution above 150 nm and larger size pores with a peak at 4.5 μm. Thermal conductivity was similar across the natural stones.

**TABLE 2:** Physical measurements of the five stone substrata employed in this study.

Substratum	Thermal Conductivity (W m^–1^ K^–1^) @ 30°C	Internal surface area (m^2^/g)	Internal pore volume (mm^3^/g)	Overall porosity (%)	Surface roughness (R_*a*_, μm)
LIMESTN	0.44	0.099	8.27	6.95	2.21
MARBLE	0.45	0.0025	0.79	0.43	1.79
SANDSP	0.40	0.98	61.87	31.91	8.07
SANDSV	0.41	2.02	6.83	19.29	6.56
GRANITE	0.45	0.13	0.96	6.28	3.30
CONCRO		4.05	19.38	4.44	25.15
CONCTN		3.79	20.12	4.63	6.81
CONCTR		3.34	21.29	4.86	11.84

Note concrete coupons were not able to be measure for thermal conductivity using our laboratory protocols/equipment.

The environmental parameters of the aquaria hosting the stone coupons during biofilm growth showed some variability for the duration of the first experiment. Air and water data logs for temperature and LUX intensity indicated measurable diurnal fluctuations of both parameters, with light levels largely consistent and water temperature levels dropping over the course of the experiment (Supplementary Figure 5). For example, there was a temperature range of 5.68°C (max: 31.78°C, min: 26.10°C) in the air temperature over the course of the experiment, and the daily mean air temperature varied by 3.10°C. The water temperature showed an overall range of 2.39°C (max: 30.05°C, min: 27.67°C). The water temperature had a maximum diurnal variability of up to 1.20°C per day. However, the mean diurnal temperature change was 0.67°C. Over the course of the experiment the mean daily temperature of the water lowered by 1.85°C.

For the metagenomic experiment, the water samples tested throughout the experiment indicate some nutrient enrichment of inorganic carbon (Table 3). In addition, there was a reduction in TIC for weeks 2 and 3. TN did not follow the same pattern of fluctuations as the carbon.

**TABLE 3:** Carbon and Nitrogen measure taken of the water present in the aquaria at the time of each sampling point.

	T_0_	Week 1 (ppm)	Week 2 (ppm)	Week 3 (ppm)	Week 4 (ppm)
TC	14.82	14.89	18.06	17.68	17.24
TIC	13.44	13.74	17.76	17.71	16.13
TOC	1.38	1.15	0.30	0.03	1.11
TN	0.13	0.08	0.10	0.07	0.15
Conductivity (ms/cm)	30.67 (0.42)	25.92 (0.55)	36.56 (0.31)	33.87 (0.37)	38.08 (0.16)
Salinity (ppt)	27.59 (0.24)	30.66 (0.46)	27.73 (0.06)	29.44 (0.05)	29.39 (0.05)
DO (mg/L)	6.08 (0.27)	8.01 (0.48)	2.99 (0.28)	4.45 (0.53)	3.53 (0.65)
ORP (mV)	126.7 (8.65)	135.53 (9.06)	183.38 (8.3)	166.13 (4.97)	154.23 (3.36)

Probe measurements of conductivity, salinity, DO, and ORP taken at the time of sampling. Standard deviation available in parenthesis. All data is in parts per million and written to 2 d.p. unless specified.

### Biofilm analyses

**FIGURE 1:**
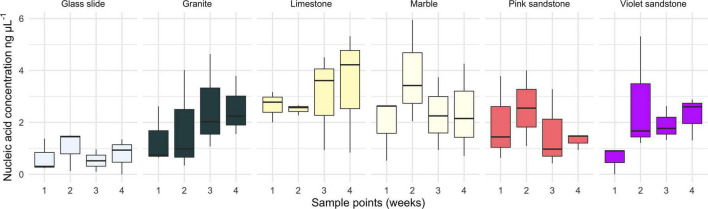
Box and whisker plot of the biofilm biomass estimates based on total nucleic acid concentration extracted from each time point grouped by substratum. Measured values were obtained using a Qubit fluorescence HS dsDNA kit.

The amplicon experiment (with time points at 3 h and 1, 3, 7, 14, and 31 days) did not yield sufficient nucleic acids to provide a confident assessment of biomass. However, the metagenomic experiment yielded measurable nucleic acid concentrations at each of the four time points (Figure 1). Data indicate that there was no significant difference in biomass among substratum types [ANOVA, *F*_(5,120)_ = 1.551, *p* = 0.179] or time points [ANOVA, *F*_(3,120)_ = 0.551, *p* = 0.649]. However, while a visual inspection of these data suggested that the biomass on glass was lower than that of calcium carbonate-based substrata (MARBLE and LIMESTN), pairwise analyses revealed no significant differences (Tukey HSD, *p* = 0.121 and *p* = 0.251, respectively).

### Amplicon sequences

**FIGURE 2:**
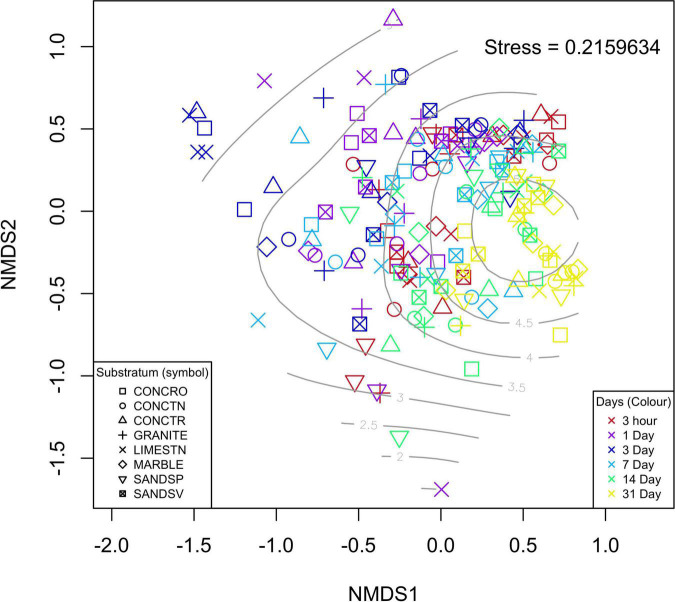
nMDS ordination of the individual samples used for 16S rRNA gene SNV analyses. Each substratum is indicated by a different symbol, whereas biofilm age is represented by color of the points on the plot. Light gray contour lines are indicative of Shannon-wiener diversity indices for each sample.

From the 192 samples processed, 190 produced suitable sequencing libraries, with two SANDSP coupons (days 3 and 31) failing to amplify. For the remaining 190 samples from the first experiment, there was no significant influence of substratum on the Prokaryotic composition based on 16S rRNA marker genes [PERMANOVA, *F*_(7,181)_ = 1.203, *p* = 0.066; Figure 2). However, the age of the biofilm resulted in significant differences in bacterial composition [PERMANOVA, *F*_(1,189)_ = 13.782, *p* = 0.001]. Pairwise investigation of these data indicates that no single substratum was significantly different in overall microbial community composition to any other substratum. Conversely, biofilm age had a significant effect on the microbial composition for all time point pairwise comparisons, with the exception of 3 h vs. 14 days and 7 days vs. 14 days which showed no statistical difference in microbial communities at these times.

Univariate analysis of the effect of biofilm age and substratum on the biofilm’s α diversity also showed no significant influence of substratum [ANOVA, *F*_(7,182)_ = 0.746, *p* = 0.634]. It was apparent that α diversity increased over time [ANOVA, *F*_(1,188)_ = 31.87, *p* < 0.001], thought to result in the similarities in β diversity observed for all samples at the end of the experiment (31 days). Analyses of the seawater controls indicated that there was no change in Bray-Curtis variability over the course of the experiment and neither did microbial diversity differ [ANOVA, *F*_(1,6)_ = 1.603, *p* = 0.29], indicating that no measurable outside influences impacted the experimental design.

The overall bacterial composition of the biofilms was dominated by a low abundance of single nucleotide variants (SNVs; Supplementary Figure 6). Most of the identified SNVs contributed < 1% of the overall relative abundance for any given sample. However, a few key taxa were dominant (high mean relative abundance) across all treatments. These included SNVs identified as *Colwellia* (5.3%), *Escherichia*-*Shigella* (4.3%), *Neptuniibacter* (3.2%), *Thalassotalea* (3.0%), and *Rhodobacteraceae* (2.9%); with *Thalassotalea* being the highest individual fraction (>80%) observed for one of the SANDSP replicates. While the overall bacterial community composition was not significantly different among substrata, 87 of the 3,631 individual SNVs identified in this study were significantly different among substratum type. These differences appeared to be primarily dominated by larger relative abundances of the SNVs on CONCRO (Supplementary Figure 7).

Examination of the 18S rRNA marker genes revealed no difference in Eukaryotic communities associated with the substratum material [PERMANOVA, *F*_(9–95)_ = 0.9125, *p* = 0.691]. There was, however, a significant difference over time within the Eukaryotic communities [PERMANOVA, *F*_(7–95)_ = 5.906, *p* = 0.001]. The 18S rRNA gene dataset yielded 4,419 specific SNVs across all treatments. From the 20 most abundant SNVs, it was clear that time was a contributing factor for their distribution (Supplementary Figure 8). From this subset (61.3% of total) of Eukaryotic SNVs, invertebrates contributed significantly to the SNVs, with *Copepoda* being responsible for > 27% of the total SNVs. Representatives of the *Choradates*, *Vertebrata* (4.53%), and *Asciidians* (4.38%) were also present. In addition, there was evidence of fungi (*Basidiomycota*; 3.48%), although the oligonucleotides used in this study were not optimized for fungal identification. In addition, the dominance of *Copepoda* 18S rRNA gene markers in the Eukaryotic fraction, is likely due to a high number of gene copy numbers within the genome his (Wyngaard et al., 1995).

### Metagenomic sequence analyses

The metagenomic experiment revealed that the metagenome and amplicon data differed with respect to the dominant taxa in the Prokaryotic and Eukaryotic groups. This difference was likely due to amplification bias from the oligos used in amplicon sequencing or the gene copy number from the rRNA gene markers monitored. For example, amplicon analyses of the Prokaryotic fraction of the biofilm indicated a dominance of *Colwellia* spp., while the metagenomic data showed that *Rhodobacterales* was the most dominant of the contigs. This was also evident in the eukaryotic fraction, with *Copeopoda* being abundant in amplicon sequences, yet absent from the common metagenome sequences. All rRNA identified from the metagenome sequencing was compared to the rRNA data from the amplicon sequencing experiments. From these data we concluded that the most abundant organisms found in the amplicon experiments were also present in the metagenome experiment, with only relative abundances differing (Supplementary Figure 9). This abundance difference was expected due to different library preparation techniques employed for the two experiments. Assessment of the alpha diversity of these *in silico* obtained rRNA genes was performed by ANCOVA and we observed that the substrata had no measurable impact [ANCOVA, *F*_(5)_ = 0.98, *p* > 0.05] on diversity, whereas the age of the biofilm did [ANCOVA, *F*_(3)_ = 3.23, *p* = 0.028]. In addition to the differences between contigs and amplicon assessments, the use of contigs enabled us to determine the relative contribution of Eukaryotic and Prokaryotic fractions. While Eukaryotic contigs were observed, their contribution was minimal compared to Prokaryotic organisms (Bacteria ∼ 98%, Archaea ∼ 0.2%, Eukaryotes ∼ 0.8%, and Viruses ∼ 0.07%; Supplementary Data—taxa abundance tables).

**FIGURE 3:**
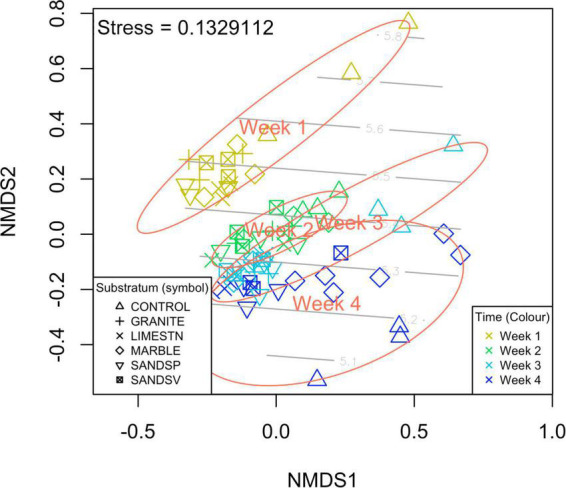
nMDS plot shows that that α and β diversity of the meta-contigs clearly separate over the *y*-axis suggesting that biofilm age is a factor of interest. Colors signify biofilm age: Gold = Week1, Green = Week2, light blue = Week 3, and dark blue = Week 4. Light gray contour lines indicate the Shannon Wiener diversity (H′).

Despite these subtle differences in biofilm community identifications, the metagenome experiment once again showed that biofilm age was a driving factor in biofilm composition [PERMANOVA, *F*_(5–71)_ = 3.45, *p* < 0.001], as was previously observed in the amplicon experiment. In contrast to the amplicon experiment, we found a significant effect of substratum on biofilm communities. However, this was only between the glass control substratum and the other stone types—there were no significant differences amongst the experimental stone types and biofilm community (Figure 3).

### Functional assessment of contigs

From the contigs there was a total of 3.9 M identified and hypothetical proteins annotated. When pooled for each treatment type across the four time points measured, these yielded 160 complete identified KEGG pathways. Of these, 81 KEGG pathways occurred within biofilms from all substrata. As with the taxonomic comparison, functional diversity also showed that the age of the biofilm had a statistically significant impact on the KEGG modules identified as complete (ANOSIM, *R* = 0.14, *p* = 0.01). However, substratum type had no significant effect (ANOSIM, *R* = 0.03, *p* > 0.05). Some KEGG modules which were common, but not shared across all samples, included Lipid A biosynthesis (M00866), Galactose degradation (M00632), and Thiosulfate oxidation (M00595). Some of the less common KEGG modules identified include Thyroid hormone biosynthesis (M00043), Glycosaminoglycan biosynthesis (M00058), and Lactosylceramide biosynthesis (M00066). As glass was the most dissimilar, significant differences in enrichment were observed for this substratum. Biofilms on glass slides were enriched for aromatic amino acid degradation while genes associated with assimilatory nitrate reduction (i.e., M00531) exhibited reduced abundance. As the biofilms increased in age, we also observed changes in the KEGG module enrichment, with initial (week 1) biofilms being enriched for salicylate (i.e., M00638). No enrichment of any KEGG module was observed for weeks 1 and 3. Four modules were enriched for weeks 1 and 4: homoprotocatechuate degradation, methane oxidation, ammonia nitrification, and comammox (i.e., M00533, M00174, M00528, and M00804, respectively). A full list of the identified modules, together with their level of completeness, can be found in Supplementary Figure 7.

## Discussion

The construction of seawalls is often the go-to defense against erosion and flooding, especially in coastal cities, hence finding ways to mitigate their negative impacts on species’ biodiversity is critical to ecological functioning and sustainable development (Firth et al., 2014; Cole et al., 2015). The current study aims to determine whether seawall construction materials affect the initial biofilm formation, with a view to identifying stone types that can provide ecological uplift. Our results show that biofilms changed, in both taxonomic composition and functional potential, over the initial 4 weeks of growth. However, these were not significantly affected by the stone type in either of the two experiments; findings that align with a recent seawall study examining colonization by macrobiota on similar materials (Hartanto et al., 2022). In contrast, smooth glass supported a different biofilm community, as compared with the natural stones. It was also observed that some SNVs were significantly more abundant on rough concrete surfaces.

The calcium carbonate-based substrata supported a consistently high biomass throughout the experiment, while the glass slides induced growth of a consistently lower biomass biofilm. This is congruent with studies describing stronger adsorption of organic matter on calcium carbonate than on silica (Carter, 1978; Zeller et al., 2020). Moreover, glass slides had an intrinsic surface roughness approximately three orders of magnitude lower (R_*a*_ ∼ 3–5 nm) than that of the stones used in this study. This difference in roughness may limit the ability of biofilms to bind strongly onto the smoother surfaces (Encinas et al., 2020). This argument is supported by the increased relative abundance of some SNVs, as found on the rougher CONCRO samples (Supplementary Figure 2). Indeed, the latter substratum harbored many of the SNVs that differed significantly among substrata. However, some studies show that larger organisms such as diatoms have the opposite relationship with surface roughness, with rough surfaces yielding lower diversity and abundance (Sweat and Johnson, 2013). Previous research from other aquatic environments, such as rivers, have also shown that surface roughness is a driver of biofilm composition and diversity (Schneck et al., 2011), as well as augmenting the biomass of benthic algae (Murdock and Dodds, 2007). Furthermore, sandstone’s substantial fraction of micron-scale porosity is expected to facilitate the penetration of filaments and enhance water retention, leading to its propensity for supporting the growth of foliose algae (Green et al., 2012).

Porosity measurements indicate pore sizes that are compatible with promoting microbial adhesion, namely above 50 nm and up to several hundred nanometers (Feng et al., 2015). Granite presented almost no pores with sub-μm size and a narrow size distribution around 4.5 μm, together with a low (≈1%) fractional porosity. LIMESTN, SANDSP, and SANDSV incorporate significant porosity, ranging over sizes from μm to sub-mm. Their interior may thus potentially harbor micro-organisms, but these were not harvested and analyzed in the present study.

Biofilm diversity (α and β) did not expose significant differences for the tested substrata, thus implying the absence of surface-induced biofilm variations. While it is probable that surface roughness and the settling organism’s size and shape may contribute to driving the recruitment process of these initial colonizers (Lu et al., 2016; Huang et al., 2018), the homogeneity in marine microbial settlement contrasts with the diverse surface chemistry of stone surfaces, as broadly classified into calcium carbonate, aluminosilicates, and concrete (Table 1). Moreover, Huggett et al. (2009) found that marine biofilm taxonomy converged on similar substrata, but that invertebrate settlement was impacted by wettability (amongst other factors). Therefore, it is likely that surface roughness, surface chemistry, and sub-surface porosity in relation to the settling organism’s size and shape are key characteristics driving the recruitment process of these initial colonizers (Lu et al., 2016; Huang et al., 2018).

The overall diversity (α and β) of the biofilms appears not to have been driven by the stone materials, but rather regulated by the age of the biofilm. In total, we observed 863 16S SNVs (2.4% of total) that where significantly different the time periods sampled. Of the SNVs most enriched were candidates from *Gemmatimonadetes*, *Calditrichaceae*, *Entotheonella*, *Microtrichales*, *Nitrsococcaceae*, *Lentisphaerae*, and four uncultured bacteria. While the average water temperature did decline throughout the experiment, this was by only ∼1.84°C (mean daily average). There was no significant change in LUX over the duration of this experiment. Data showed (Figure 2) that, as the biofilms age, α diversity indices increased and the dissimilarly between biofilms on each substratum (β diversity) reduced, in the case of amplicon sequences. This increase in α diversity and decrease in β diversity is also observed in similar on-going field experiments in the region (data unpublished). However, the α diversity of the metagenome samples only showed minor fluctuations. This is most likely due to the lack of high frequency sampling in the first weeks, such as was employed for the amplicon experiments. This indicates a more homogenous biofilm with increased convergence in identified SNVs and identified contigs. For the contigs the α diversity was most similar at weeks 2 and 3, which matched the reduction in TOC measures. This suggests that recruitment from the water column may be a factor in regulating the α diversity. The β diversity changes on the glass controls (open triangles in Figure 3) were subtly different from other substrata of an equal age, supporting the observation that the (sub-nm) roughness of the glass may be more important than the chemical composition of the glass when compared to the silicate stones (SANDSP and SANDSV).

In addition to taxonomic diversity, we examined functional traits. Given the complexity in microbial systems, and that functional diversity does not have a global definition (Escalas et al., 2019), we opted for trait-based richness as a measure of functional potential. We showed that the age of the biofilm was a driver for changes in functional trait richness, although *post hoc* analyses show that it was only samples from weeks 1 to 3 that differed significantly. Both the functional and taxonomic differences observed could also be influenced by lower TOC levels at week 3, which may have altered recruitment. However, our data do not determine this conclusively (Supplementary Figure 10).

By observing enrichment of functional trait richness, the differences between the time points was associated with enrichment of specific KEGG modules only for weeks 1 and 4. This enrichment could have implications with respect to the function of the biofilm community and the efficacy by which invertebrate larvae settle on the biofilms. For example, the presence of methanotrophs in weeks 1 and 4 may reduce the settlement of some coral larvae as it has been shown that the presence of methanotrophs deter larval settlement (Sneed et al., 2015). Nevertheless, Petersen and Dubilier (2009) showed that methanotrophy can encourage symbiosis between invertebrates and bacteria, suggesting that not all invertebrates would be repelled by these bacteria. A similar scenario is also likely for coral settlement with regards to nitrogen fixation, as N_2_ is a major factor in coral health (Rädecker et al., 2015) and any changes in the nitrogen cycling ability of the biofilm may impact the settlement of coral larvae. Therefore, the enrichments observed for weeks 1 and 4, as well as the reduction observed on the glass substrata, could impact what types of invertebrate larvae choose to settle on these surface biofilms. Furthermore, the increase in biomass over time observed for some of the substrata may further enhance invertebrate settlement as it has been shown that increases in settlement occur with high bacterial cell numbers (Lema et al., 2019). However, it is unclear from these data whether this was an influence of biomass alone or a change in function that would likely result from a higher cell density within the biofilm.

## Conclusion

The initial aim of this study was to determine what effect different seawall building material would have on the taxonomic and functional diversity of a typical marine biofilm during early settlement. We discovered, however, that differences in material surface chemistry and topography had no measurable lasting impact on the biofilms. Only glass, which was orders of magnitude smoother, and the rougher concrete substratum, had any significant influence on the biofilm composition, and only during the initial stages. The overall observation was that the marine biofilms converged over time to a generic marine biofilm, rather than the underlying stone substrata type playing a significant role in driving distinct communities.

## Data availability statement

The datasets presented in this study can be found in online repositories. The names of the repository/repositories and accession number(s) can be found in the article/Supplementary Material.

## Author contributions

SS, YP, DM, PT, WB, and SR equally contributed to the design of this project. SS, YP, and DV generated all data and performed analyses. All authors contributed to the writing of this article.
