# Supplementary material for: Bacterial biofilm colonization and succession in tropical marine waters are similar across different types of stone materials used in seawall construction
Source: Front Microbiol. 2022 Jul 25;13:928877. doi: 10.3389/fmicb.2022.928877 (PMC9358718; doi:10.3389/fmicb.2022.928877)
Supplement: Supplementary file 6 [file Data_Sheet_6.docx]

Supplementary Figures

(A)


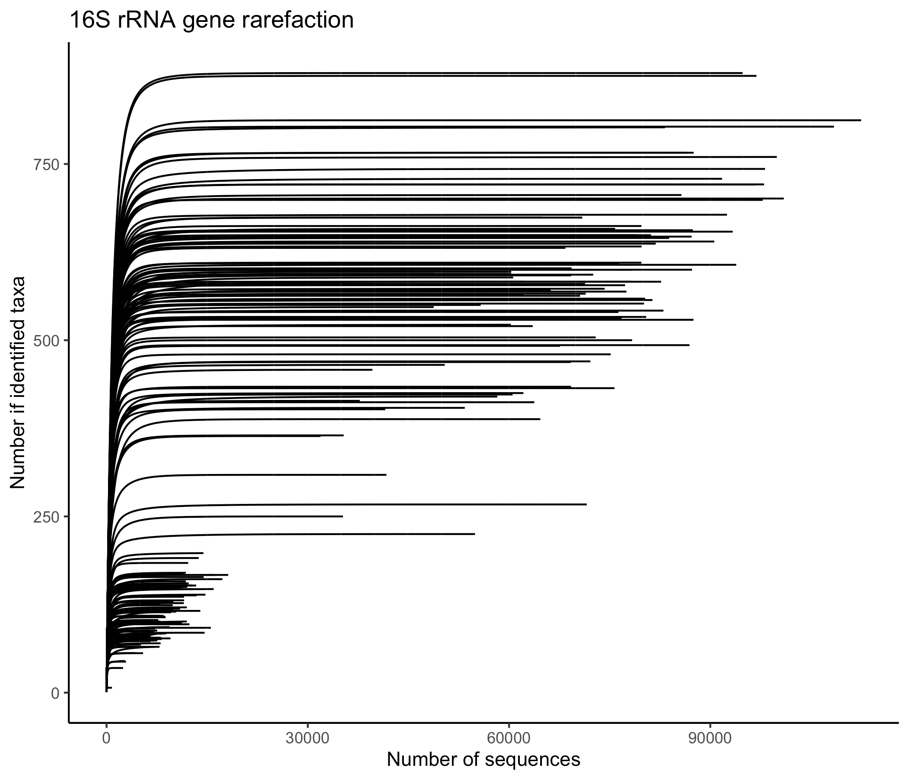


(B)


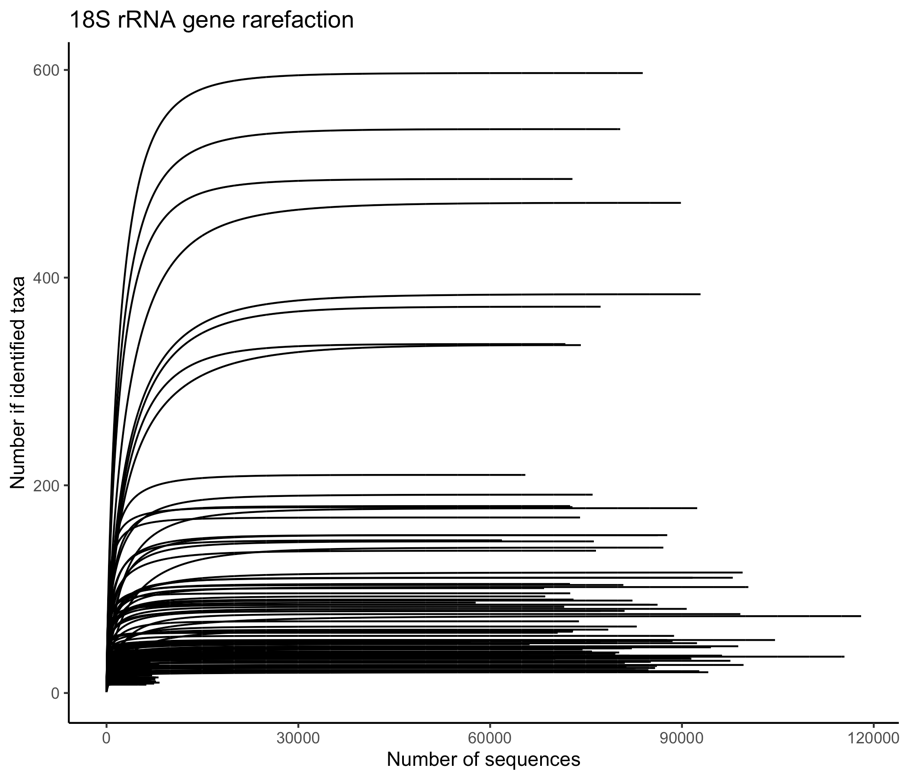


Suppl Figure 1. Rarefaction plots for all 16S rRNA genes (A) and 18S rRNA genes (B) identified and employed in the amplicon experiments. These lines represent the species richness of all samples.


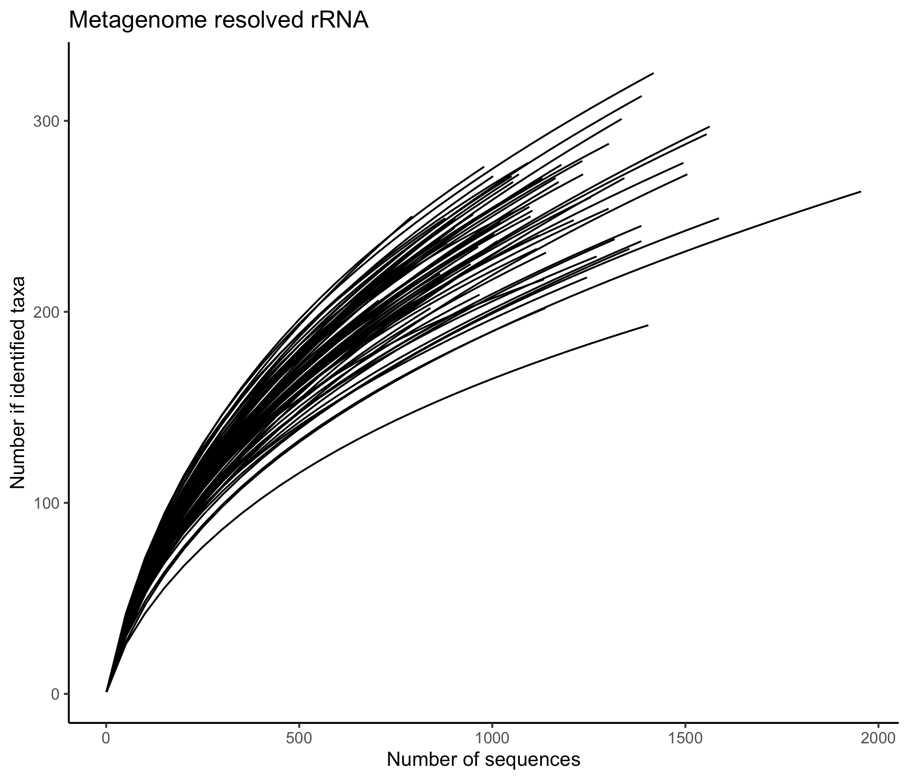


Suppl Figure 2. A rarefaction plot for all rRNA genes identified from the metagenomic experiment. These lines represent the species richness of all samples. All rRNA genes were identified from contigs using “sortmerna” and the Silva 132 database.


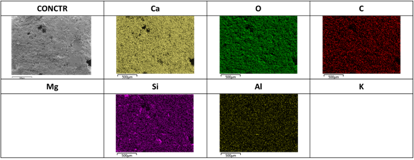

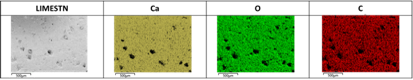

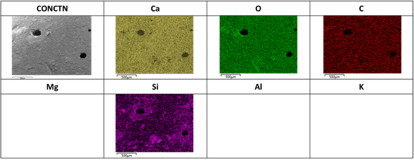

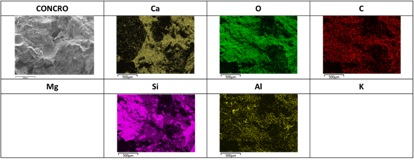


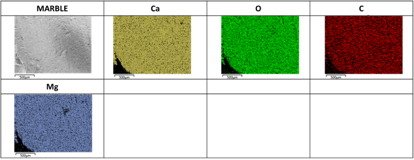

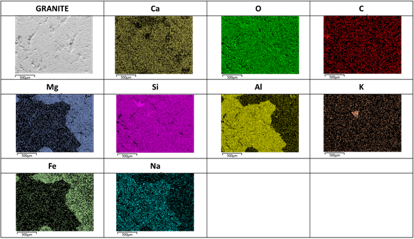


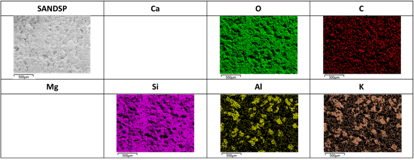


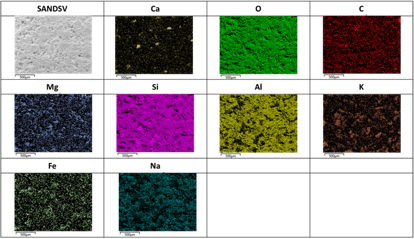


Suppl Figure 3. Surface elemental maps of all treatment stone types, as obtained from SEM/EDX, for elements > 1 % w/w. The coloured surface elemental maps correspond to the greyscale SEM image in the top left cell. Colour intensity in the elemental maps reflects the location and quantity of each element specified, with black areas indicating either surface pores or an absence of the element, and brighter regions indicating a higher percentage of the element. Scale bars represent 500μm


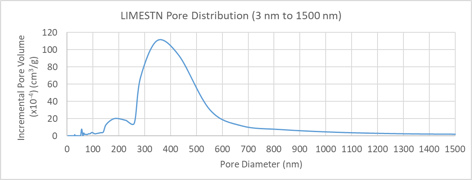

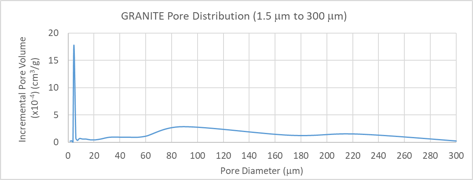

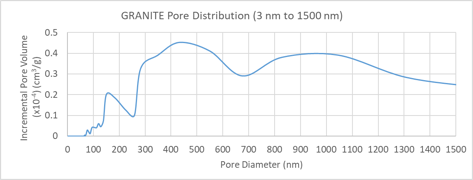

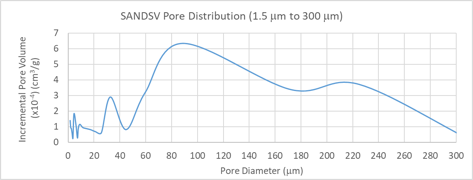

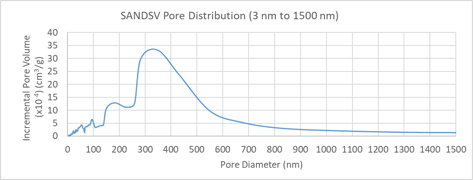

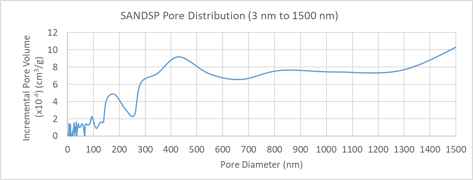

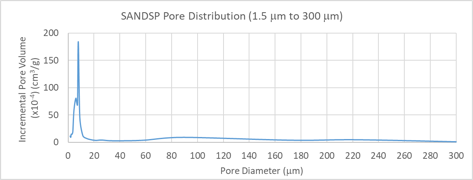

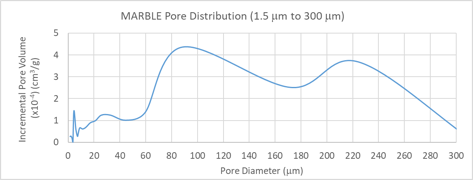

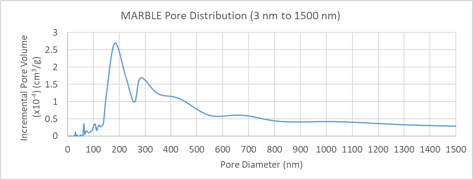

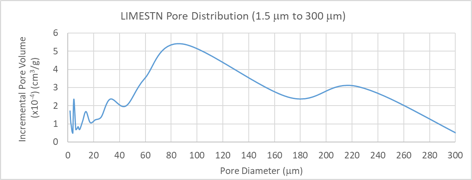


Suppl. Figure 4. Pore size distribution from 3 nm to 300 μm of natural stones: a) limestone, b) marble, c) pink sandstone, d) purple sandstone and e) granite, as obtained from mercury intrusion porosimetry. Please note that the axes in these plots are not fixed and therefore visual comparisons are not possible.

b) ii)

e) ii)

e) i)

d) ii)

d) i)

c) ii)

c) i)

b) i)

a) i)

a) ii)


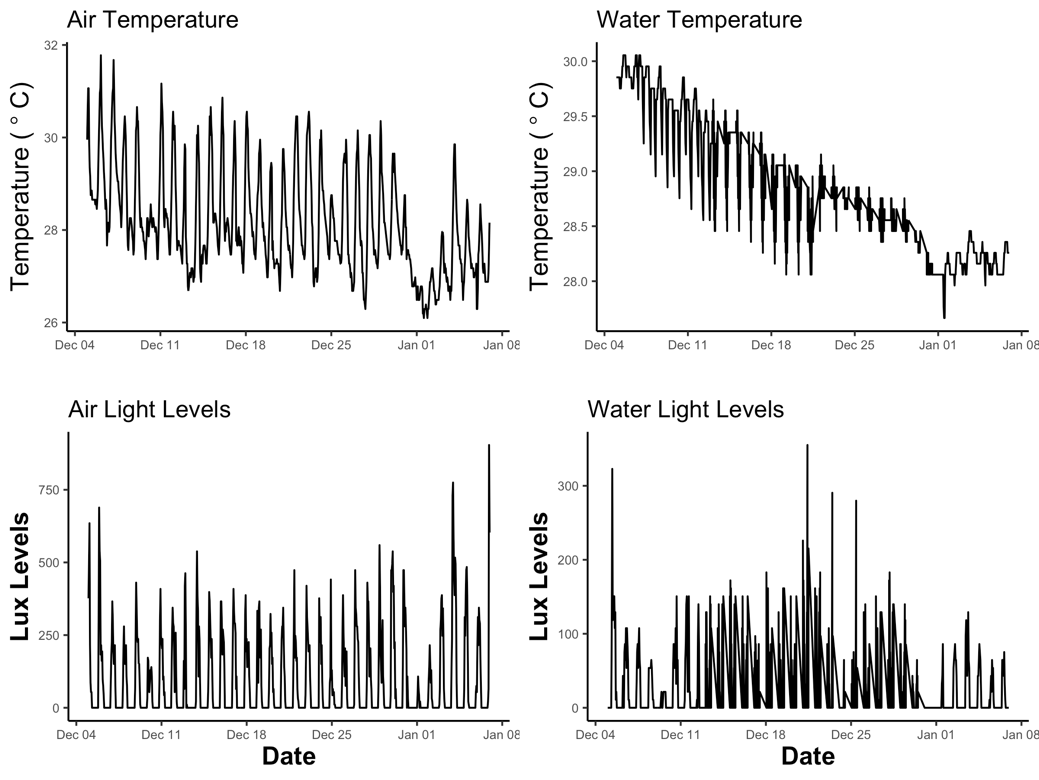


Suppl. Figure 5. Diurnal fluctuation in temperature (˚C) and LUX intensity for both the air and water environments of the aquaria in which the stone coupons were incubated for 31 d. All data was collected using Hoba data loggers deployed with substratum for the duration of the experiment.


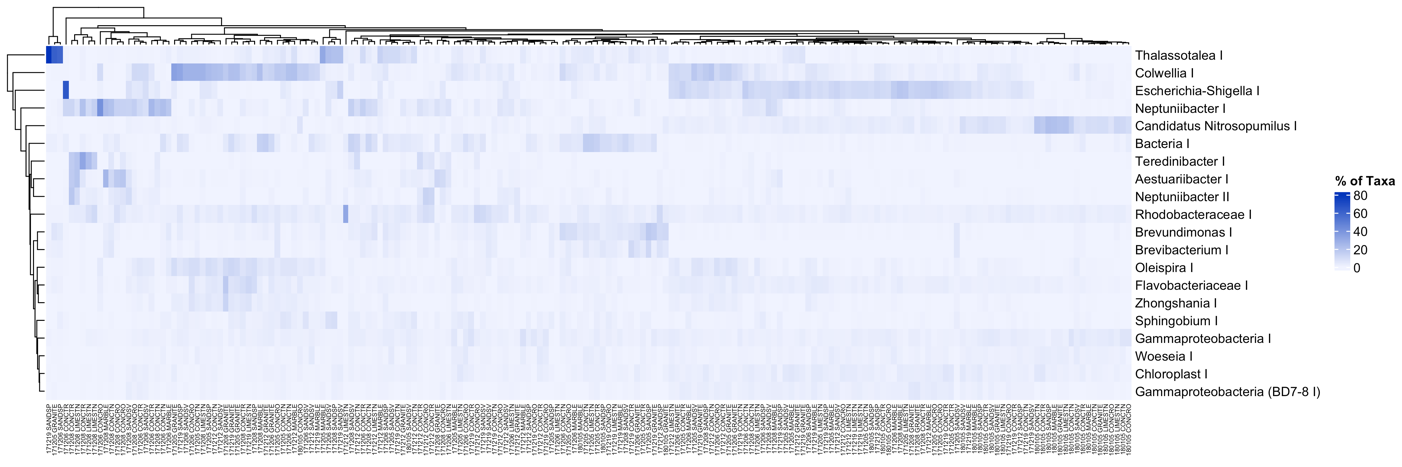


Suppl Figure 6. Heatmap showing the top 20 most abundant SNVs identified from the 16S rRNA amplicon sequencing analyses. The x axis labels indicate the type of material and the date of collection. dendrograms formed using nearest neighbour similarity. Axis labels given as “Substrata – date (y/m/d) – rep”.


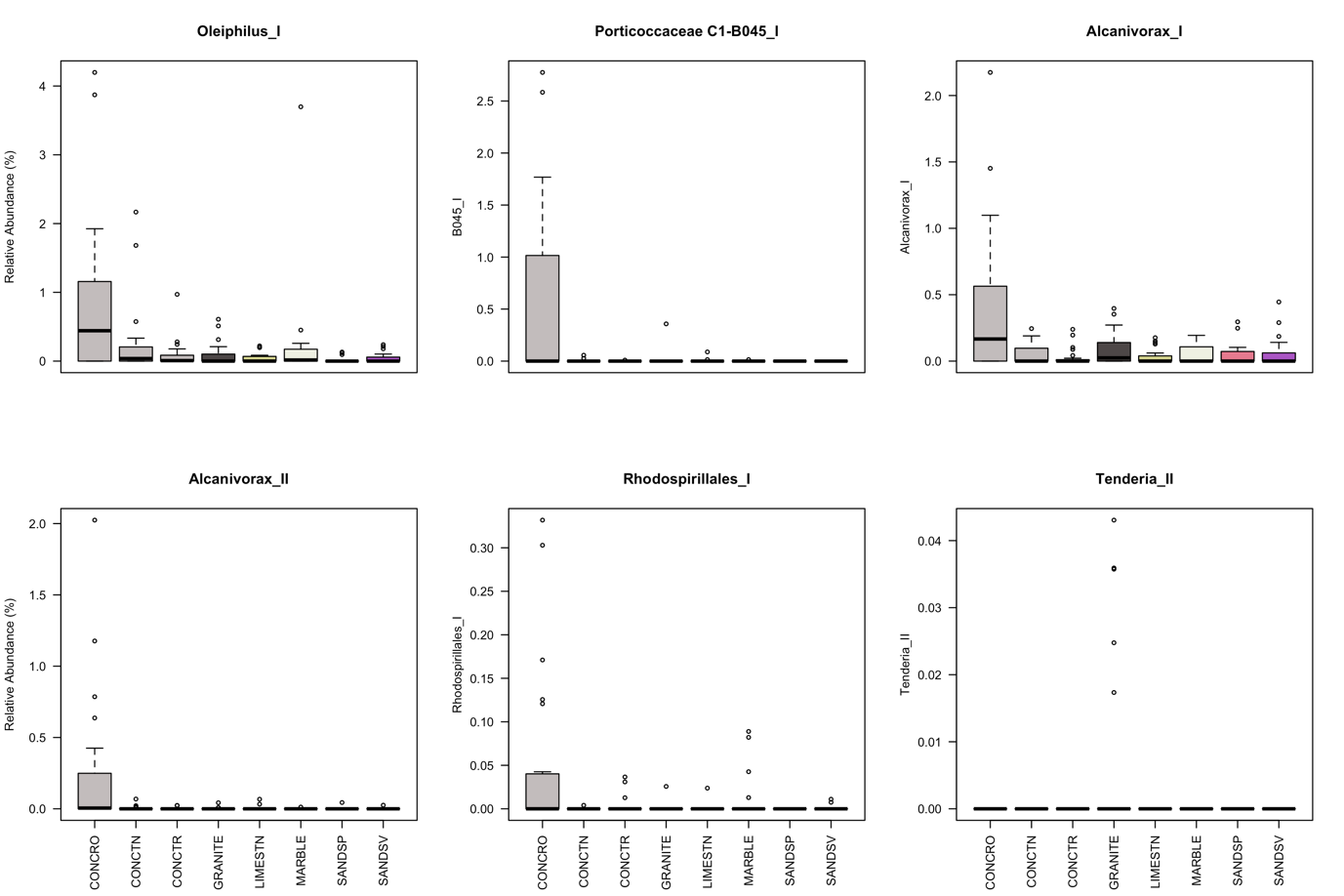


Suppl. Figure 7. From the 87 SNVs identified, within the amplicon experiment, as significantly differing across the substrata samples, the six SNV with lowest p-values were selected to display what these differences were. Relative abundance of the SNVs can be seen across all eight of the substrata used in the amplicon experiment.


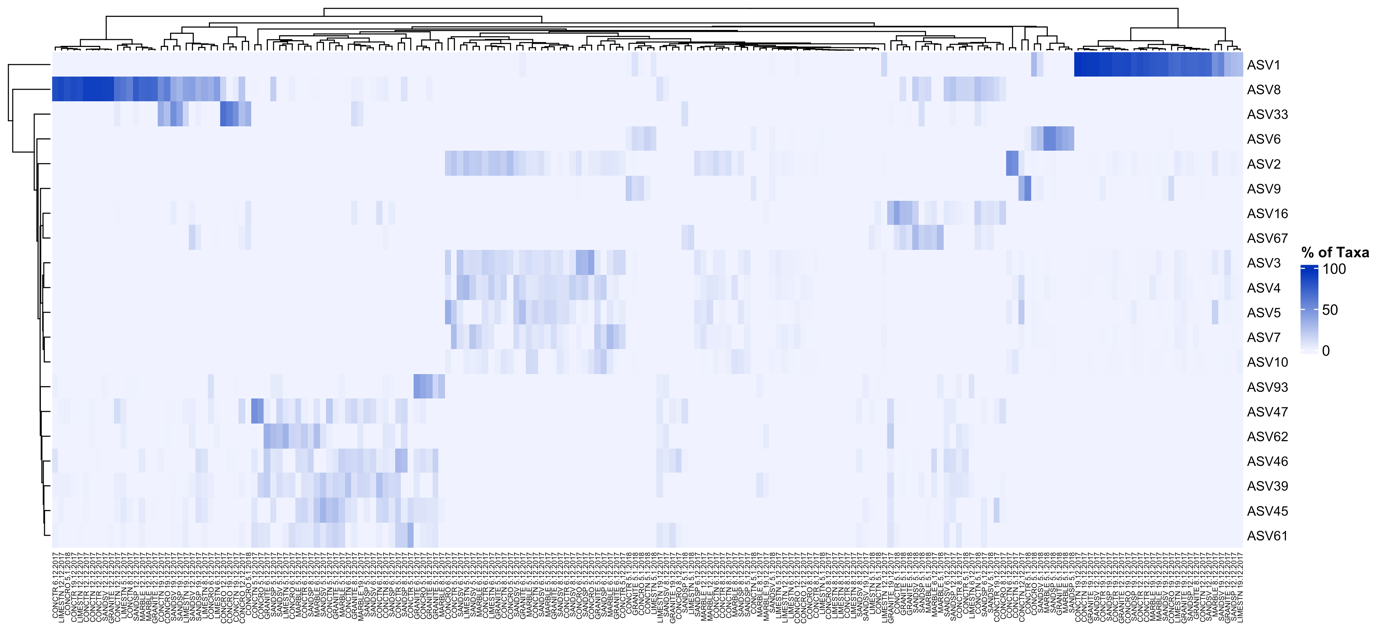


Suppl Figure 8. Heatmap showing the top 20 most abundant SNVs identified from the 18S rRNA amplicon sequencing analyses. The x axis labels indicate the type of material and the date of collection. Dendrograms formed using nearest neighbour similarity. Axis labels given as “Substrata – date (d/m/y)”.


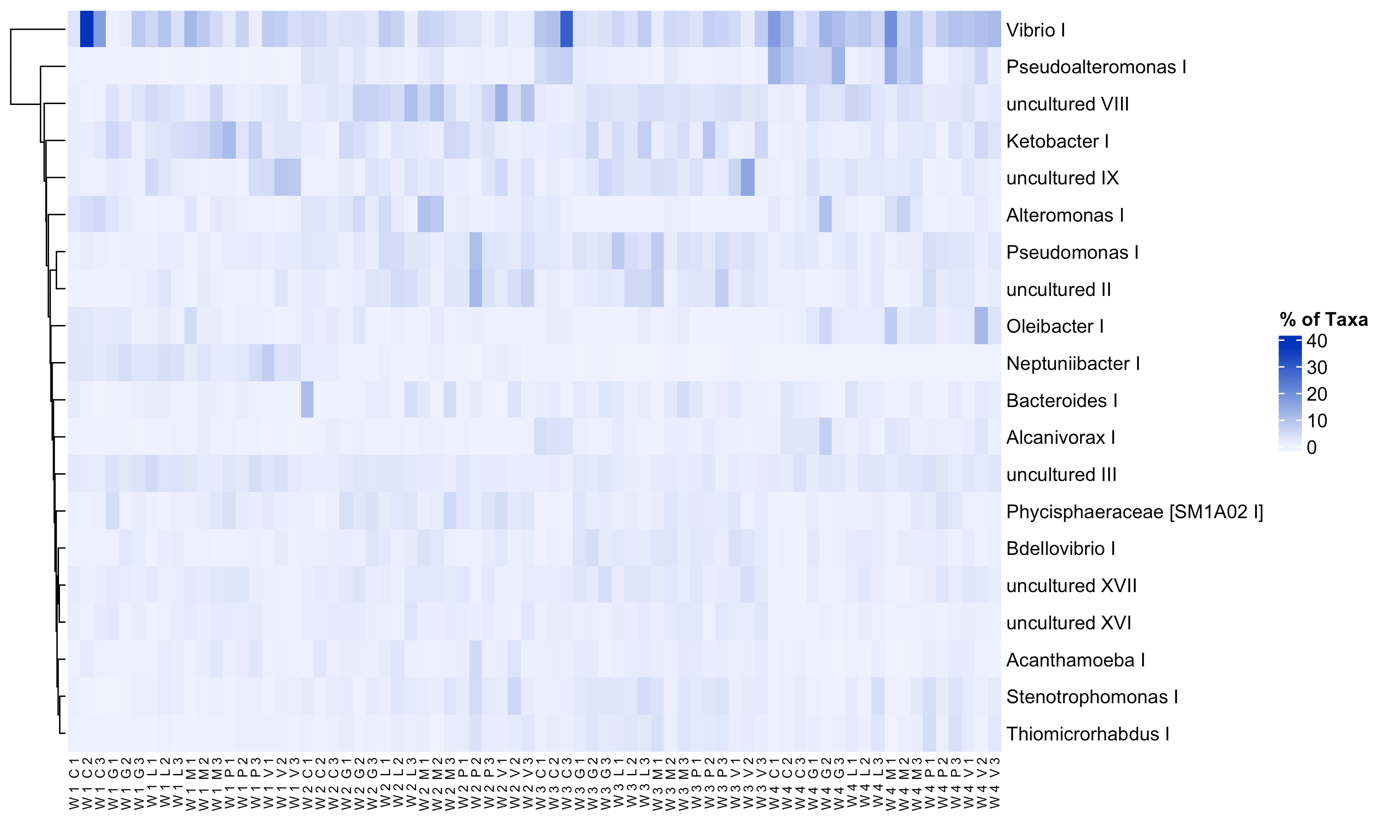


Suppl Figure 9. Heatmap showing the top 20 most abundant taxa identified from the rRNA *in-silico* identified from the metagenome data . The x axis labels indicate the type of material and the date of collection. Dendrograms formed using nearest neighbour similarity. Axis labels given as “week (i.e. W 2) and substrata (i.e. V 2)”. C = Glass Control, L = Limestone, M = Marble, P = Pink sandstone, and V = Violet sandstone.


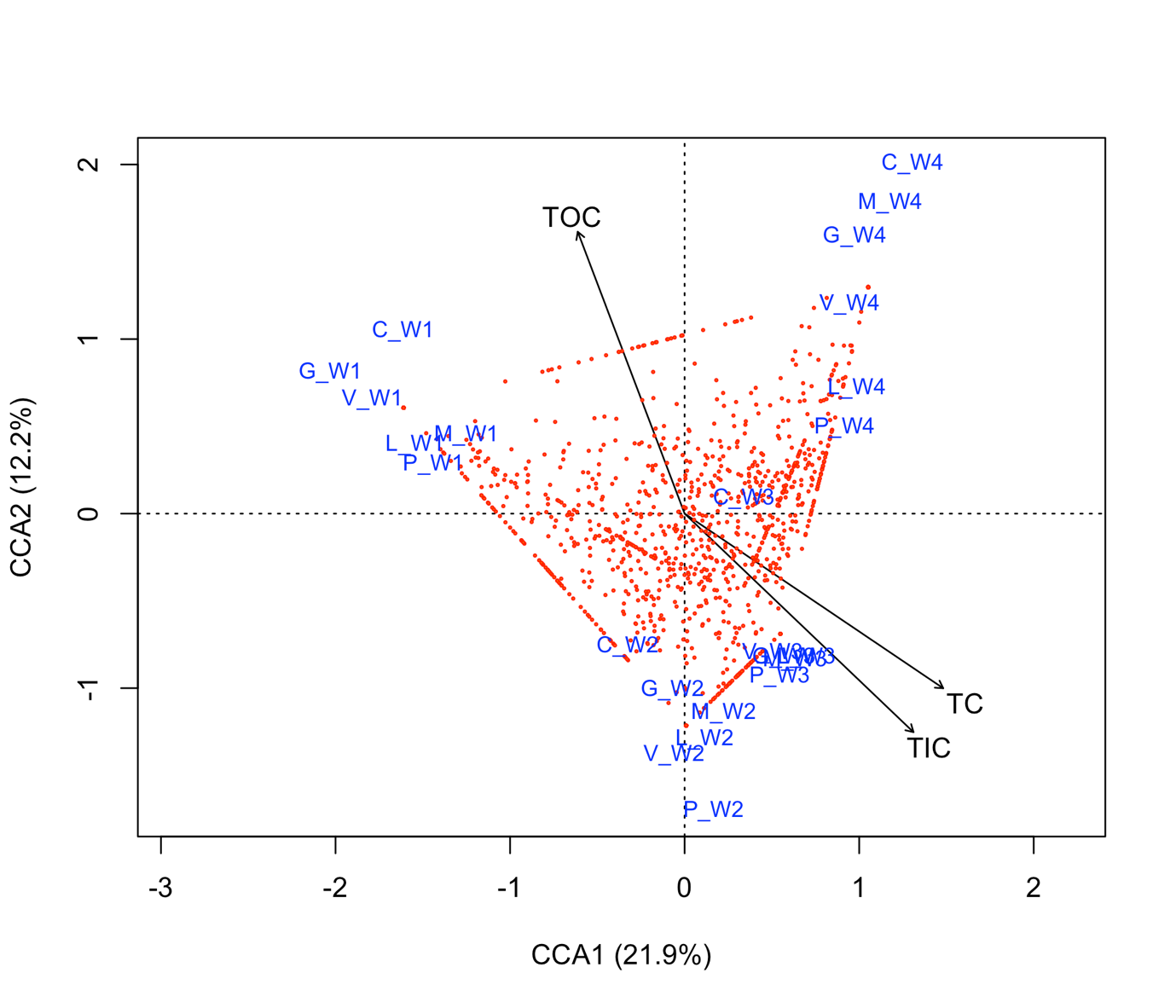


Suppl Figure 10. A CCA plot of the metagenome sequence data overlaid with physiocochemical data from table 3. The lower TOC offered an inverse-correlation with the diversity of the biofilms.
