# Supplementary figures and images for: Bacterial biofilm colonization and succession in tropical marine waters are similar across different types of stone materials used in seawall construction
Source: Front Microbiol. 2022 Jul 25;13:928877. doi: 10.3389/fmicb.2022.928877 (PMC9358718; doi:10.3389/fmicb.2022.928877)

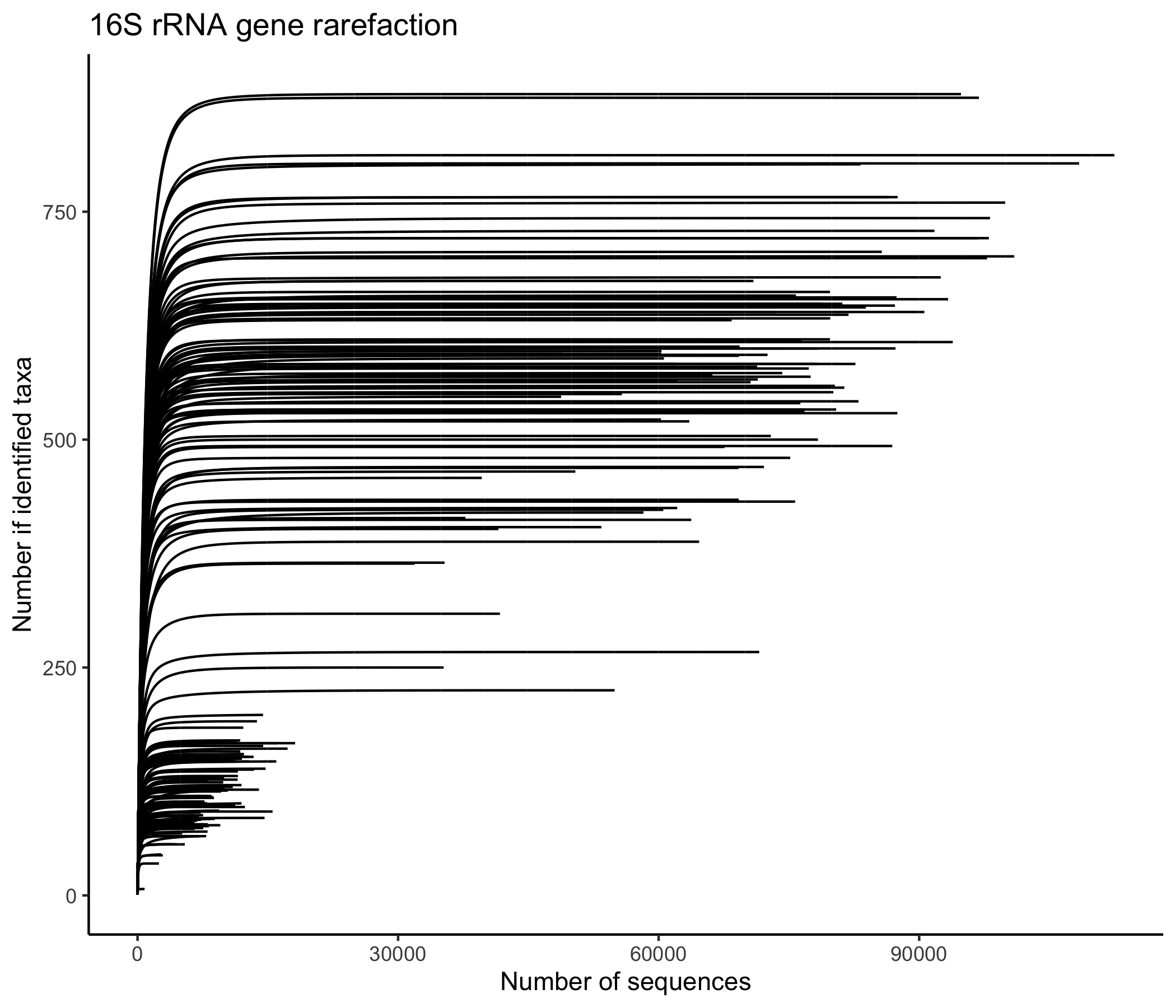

Supplement: Supplementary file 7 [file Image_1.TIFF]

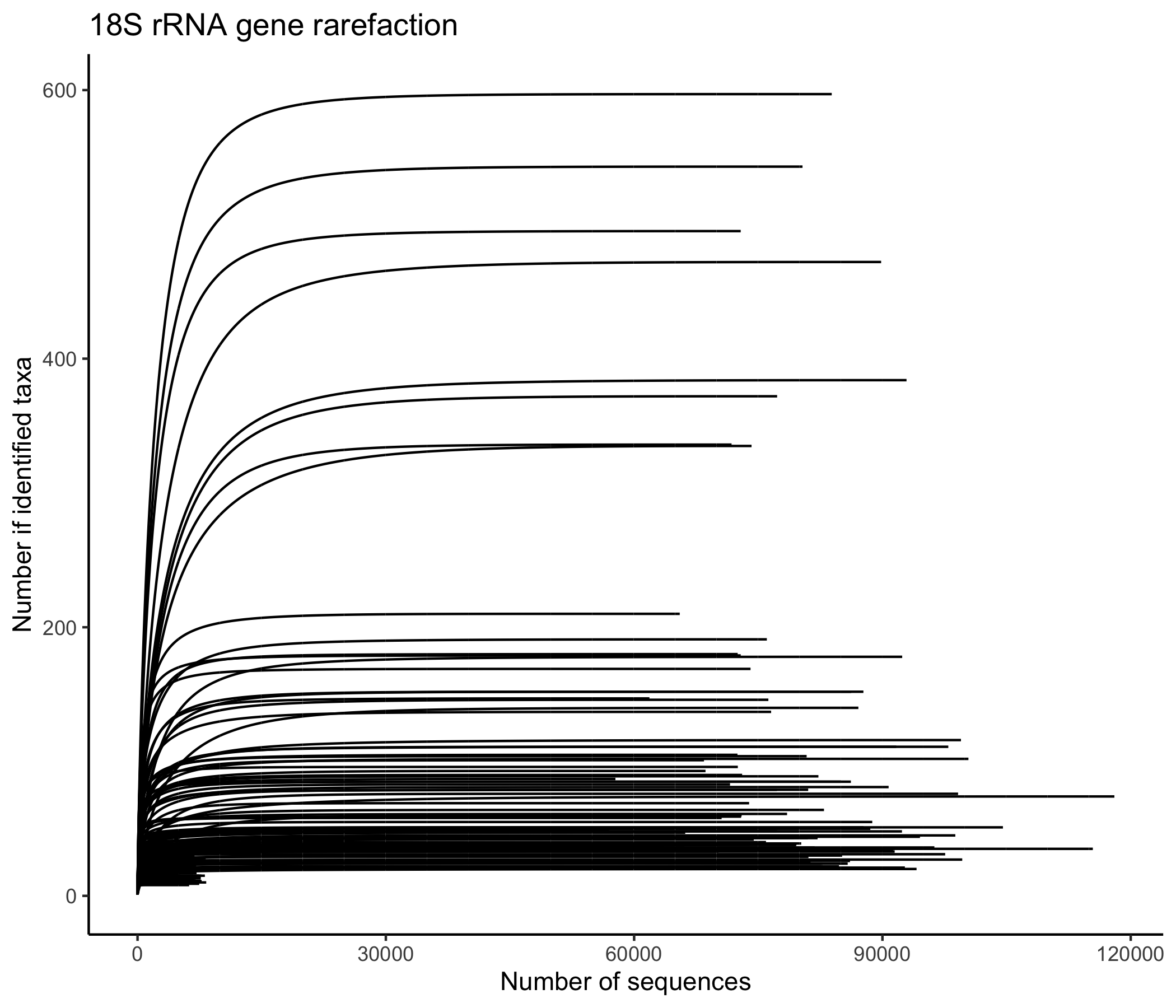

Supplement: Supplementary file 8 [file Image_2.TIFF]

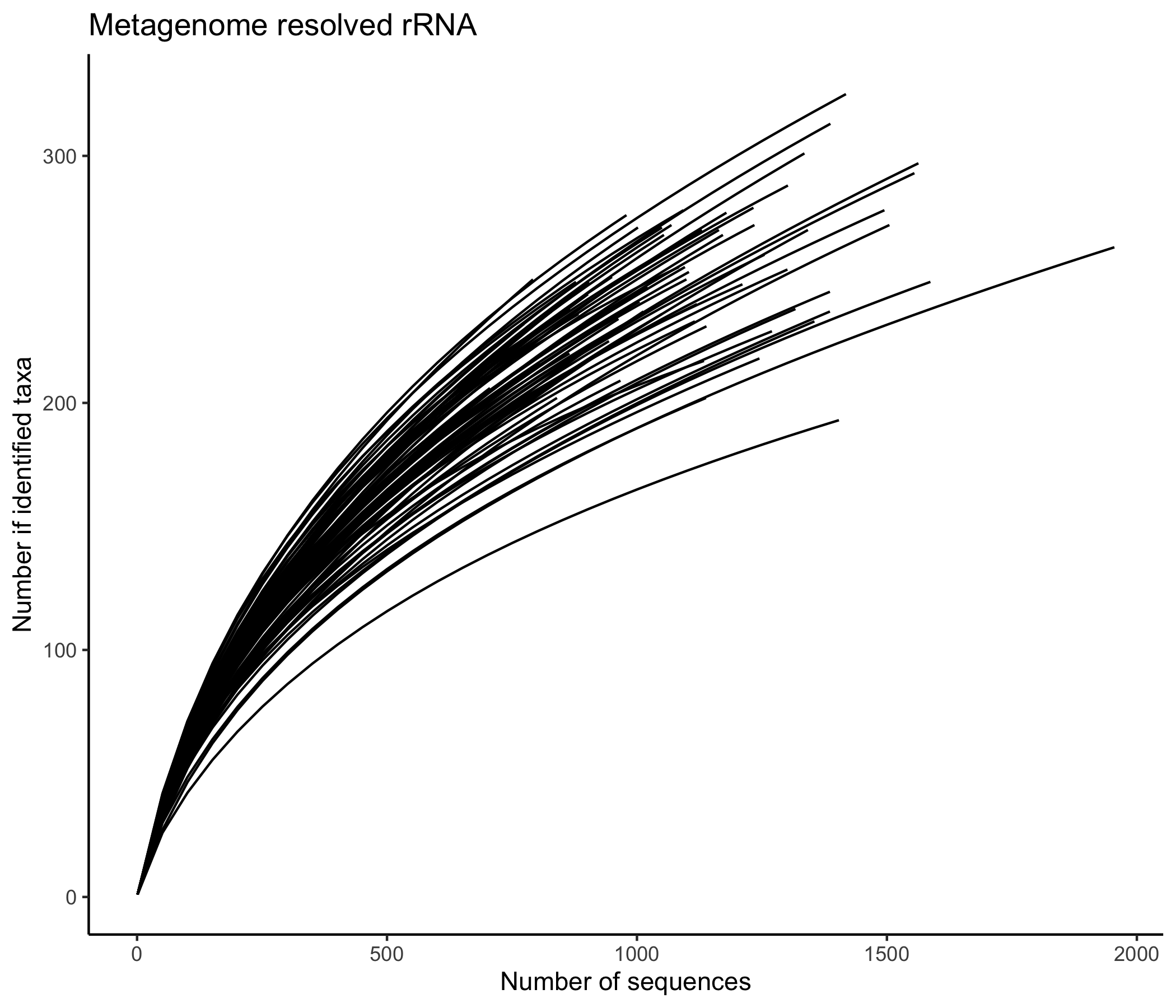

Supplement: Supplementary file 9 [file Image_3.TIFF]

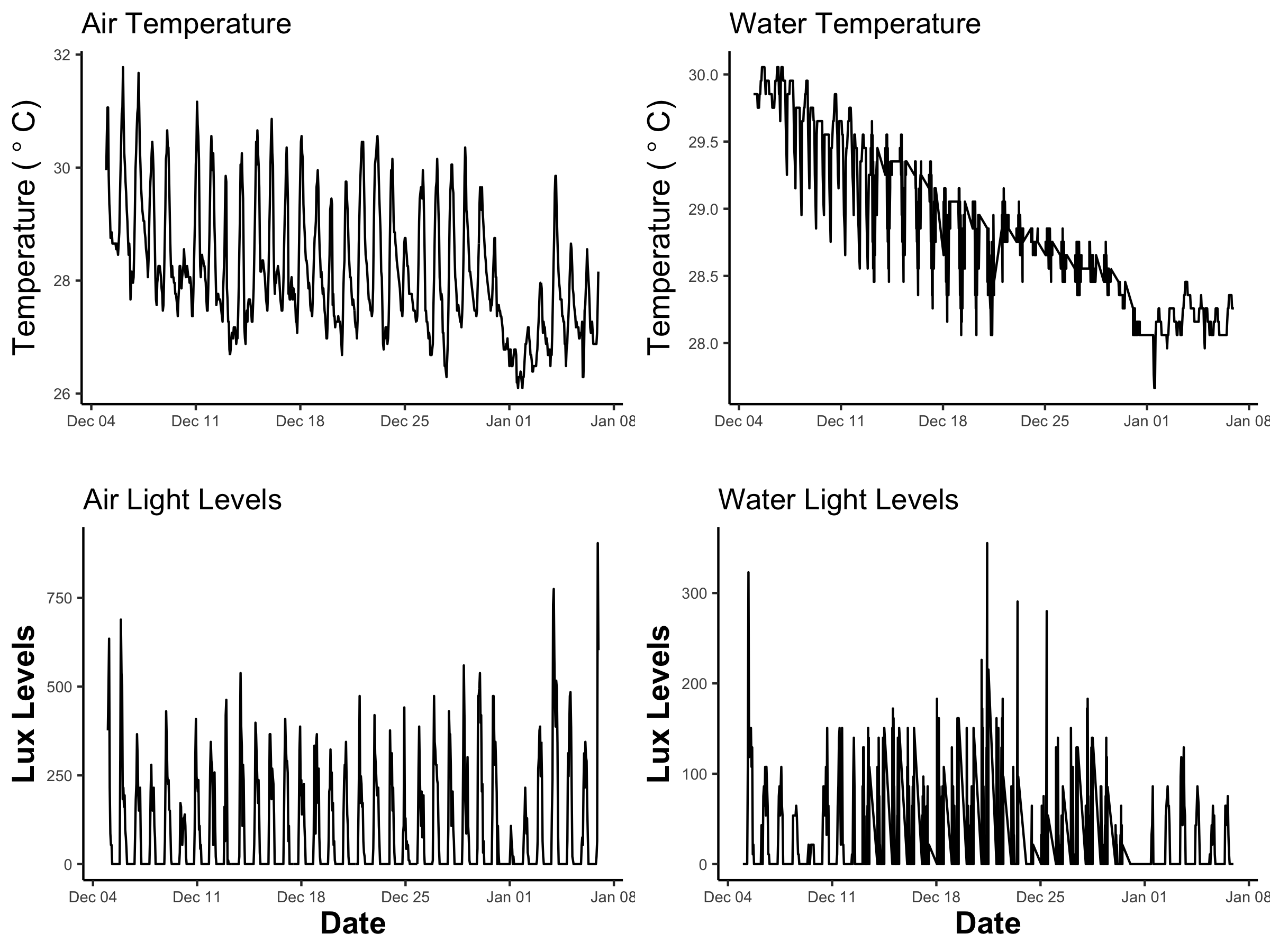

Supplement: Supplementary file 10 [file Image_4.TIFF]

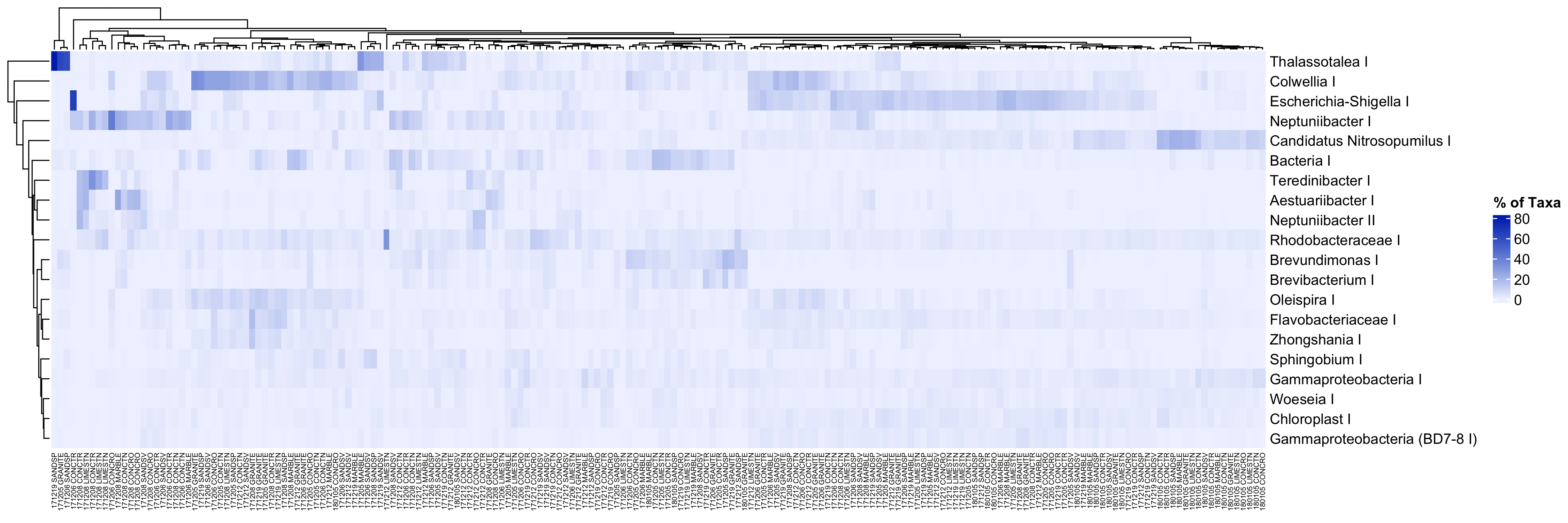

Supplement: Supplementary file 11 [file Image_5.TIFF]

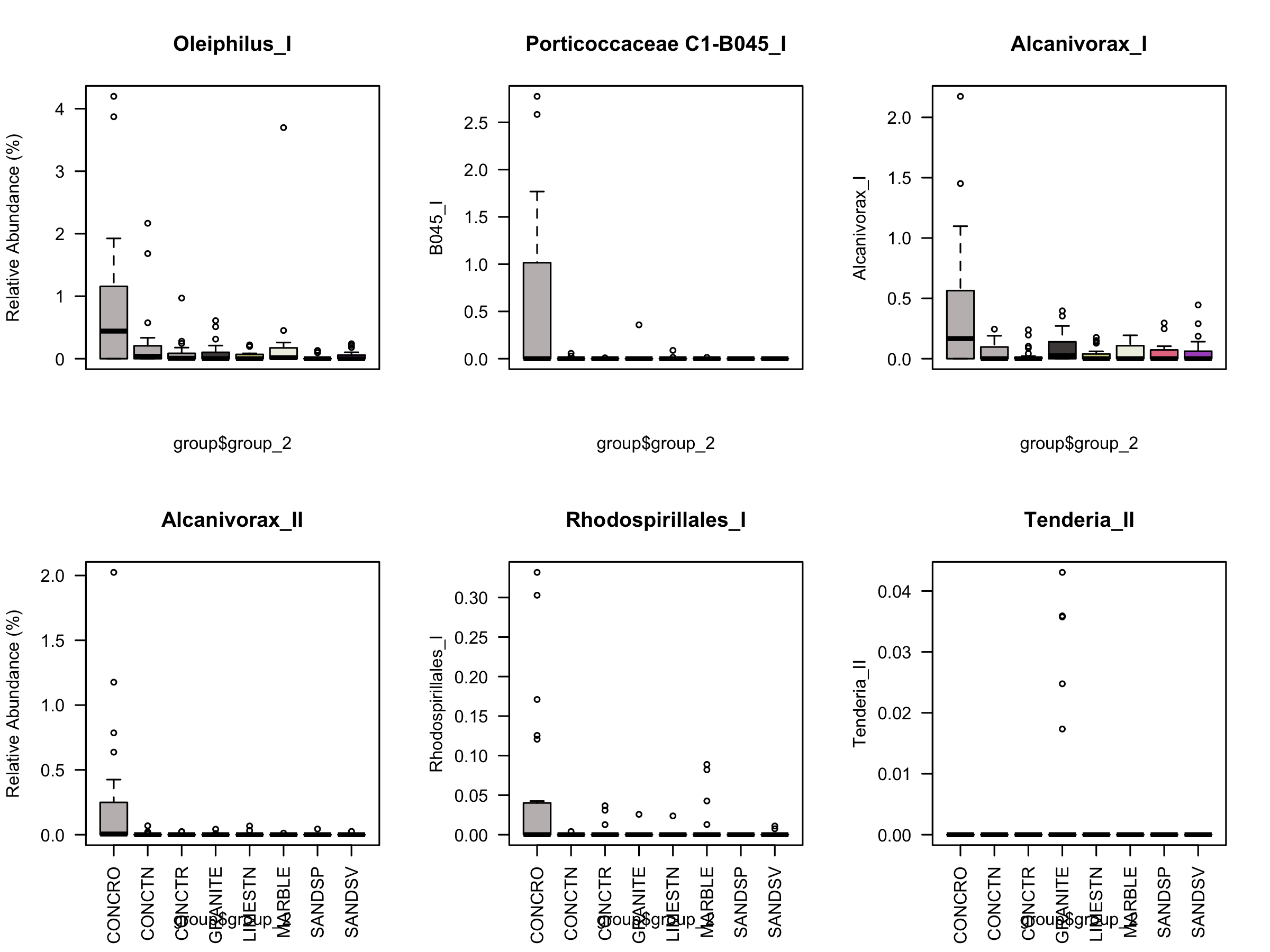

Supplement: Supplementary file 12 [file Image_6.TIFF]

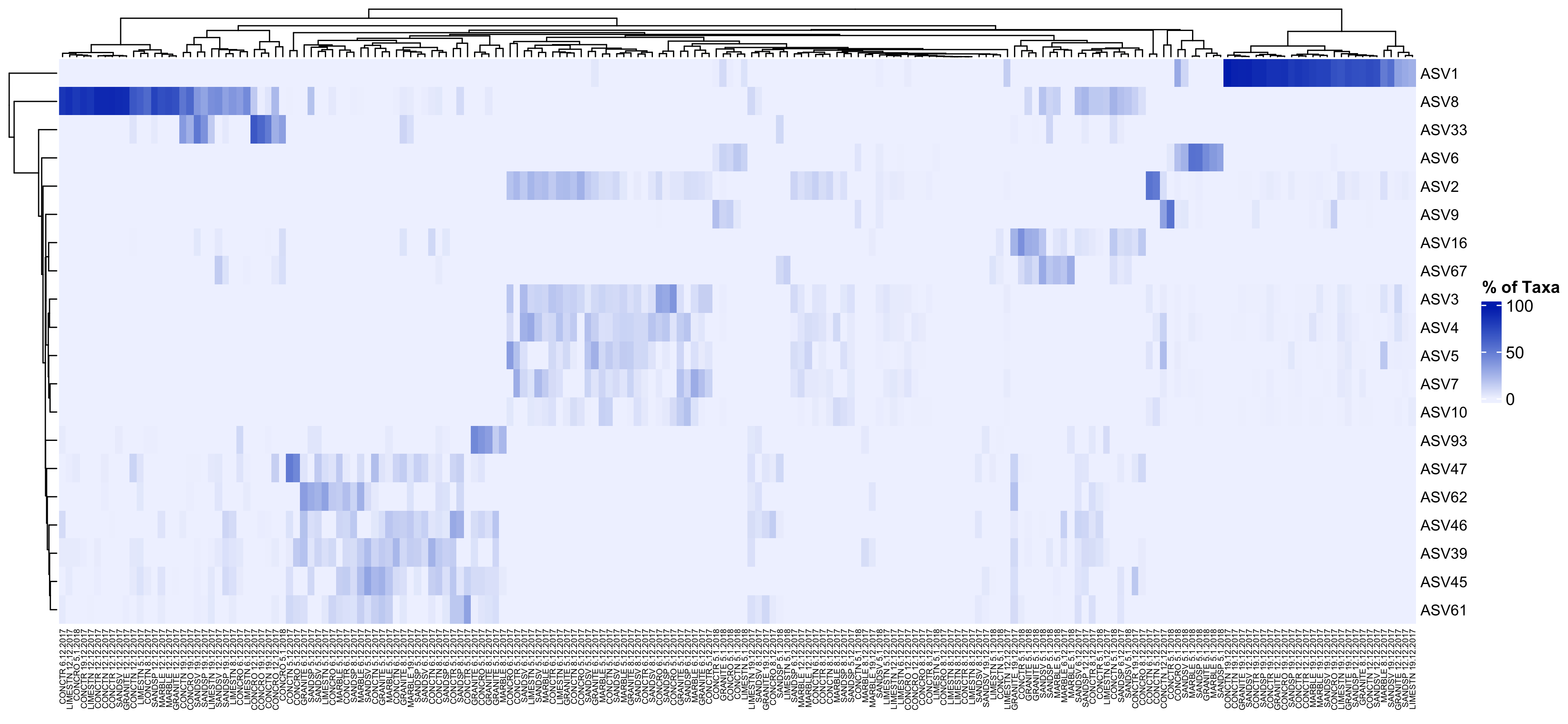

Supplement: Supplementary file 13 [file Image_7.TIFF]

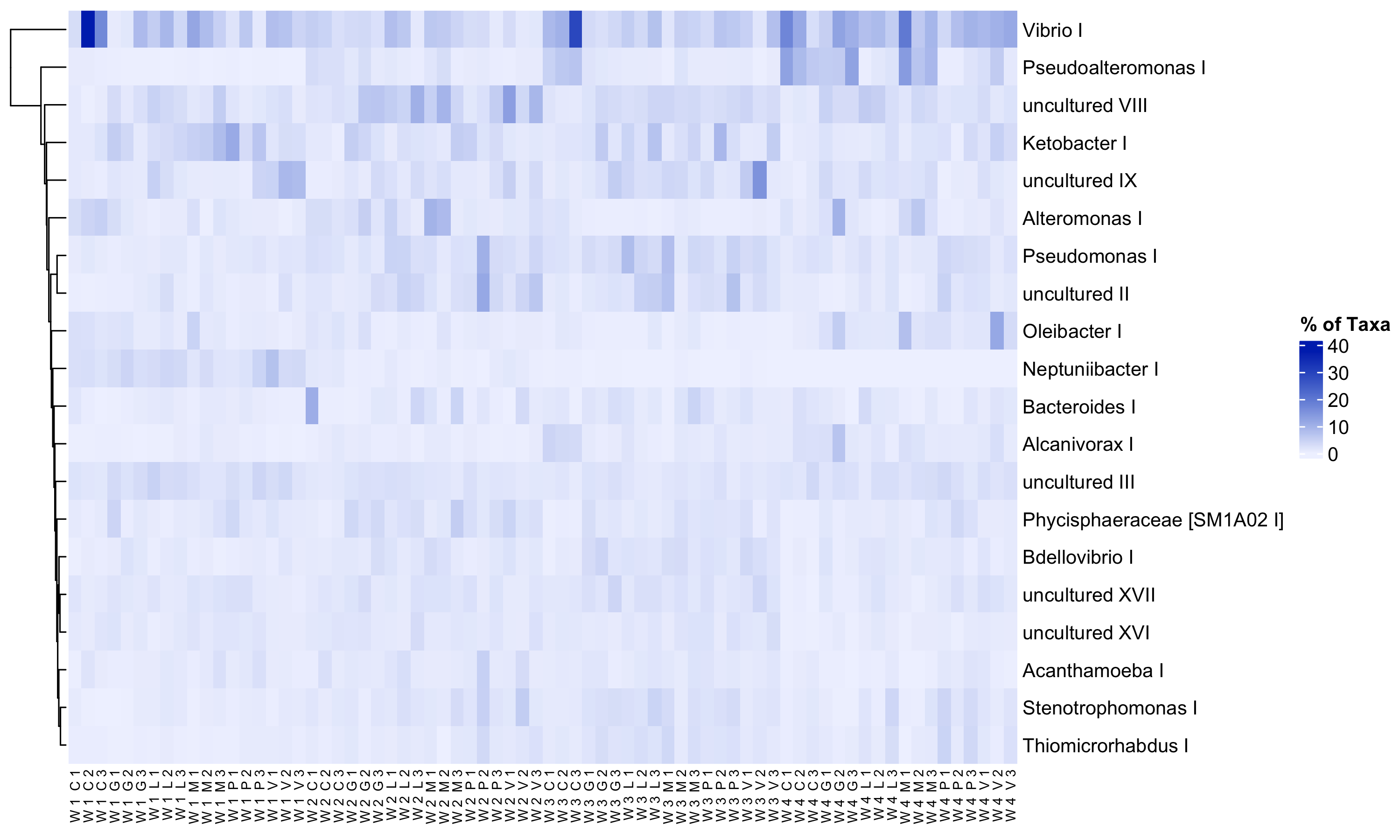

Supplement: Supplementary file 14 [file Image_8.TIFF]

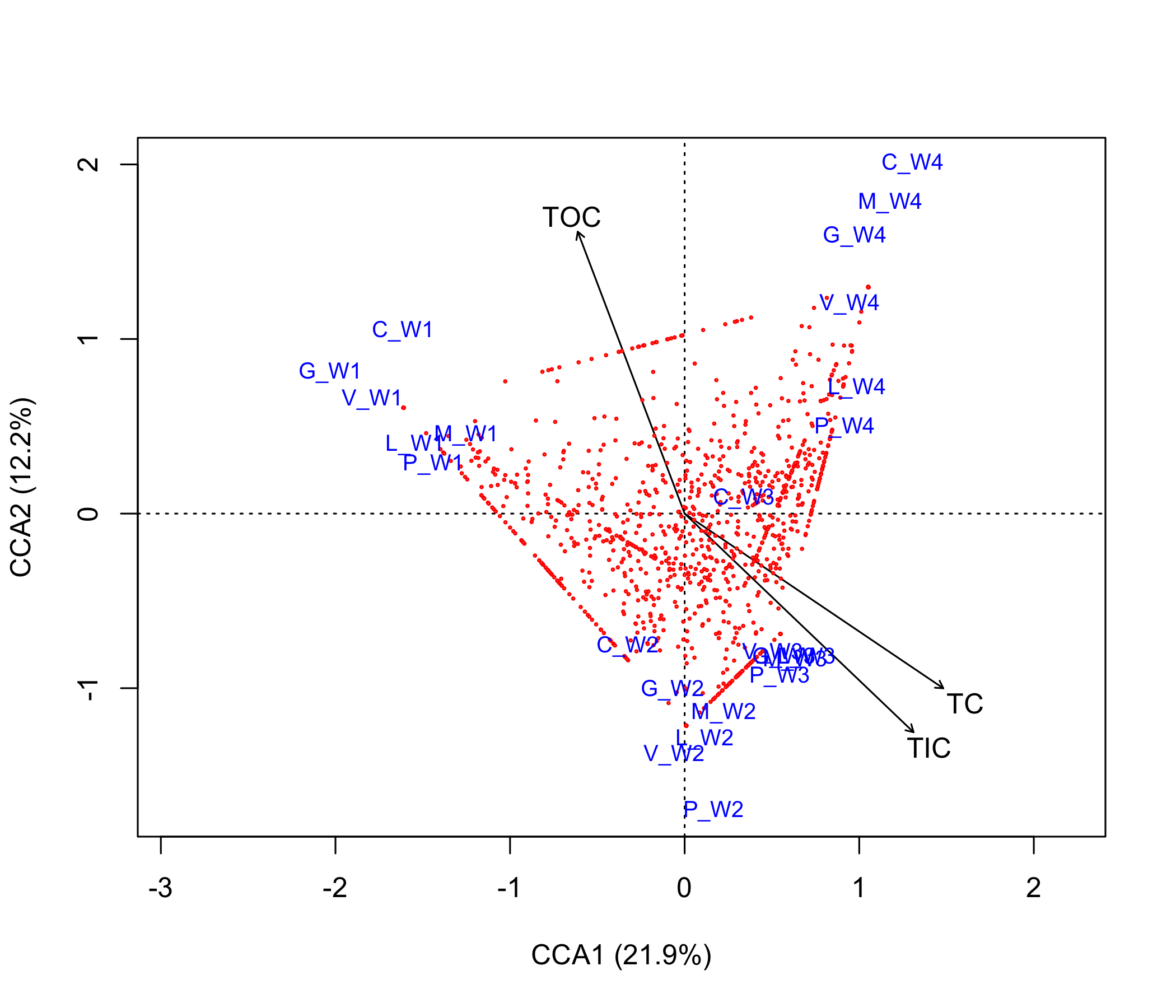

Supplement: Supplementary file 15 [file Image_9.TIFF]
